# The impact of non-alcoholic fatty liver disease and liver fibrosis on adverse clinical outcomes and mortality in patients with chronic kidney disease: a prospective cohort study using the UK Biobank

**DOI:** 10.1186/s12916-023-02891-x

**Published:** 2023-05-18

**Authors:** Theresa J. Hydes, Oliver J. Kennedy, Ryan Buchanan, Daniel J. Cuthbertson, Julie Parkes, Simon D. S. Fraser, Paul Roderick

**Affiliations:** 1 Department of Cardiovascular and Metabolic Medicine, 3Rd Floor Clinical Sciences Centre, Institute of Life Course and Medical Sciences, Liverpool University Hospitals NHS Foundation Trust, University of Liverpool, Longmoor Lane, Liverpool, L9 7AL UK; 2 University Hospital Aintree, Liverpool University Hospital NHS Foundation Trust, Liverpool, L9 7AL UK; 3 School of Primary Care, Population Sciences and Medical Education, University of Southampton, Southampton, SO17 1BJ UK; 4 Department of Hepatology, University Hospitals Southampton NHS Foundation Trust, Southampton, SO16 6YD UK; 5 NIHR Southampton Biomedical Research Centre, Southampton General Hospital, Southampton, SO16 6YD UK

**Keywords:** Non-alcoholic fatty liver disease, Chronic kidney disease, Cardiovascular disease, Multi-morbidity

## Abstract

**Background:**

Chronic kidney disease (CKD) and non-alcoholic fatty liver disease (NAFLD) frequently co-exist. We assess the impact of having NAFLD on adverse clinical outcomes and all-cause mortality for people with CKD.

**Methods:**

A total of 18,073 UK Biobank participants identified to have CKD (eGFR < 60 ml/min/1.73 m^2^ or albuminuria > 3 mg/mmol) were prospectively followed up by electronic linkage to hospital and death records. Cox-regression estimated the hazard ratios (HR) associated with having NAFLD (elevated hepatic steatosis index or ICD-code) and NAFLD fibrosis (elevated fibrosis-4 (FIB-4) score or NAFLD fibrosis score (NFS)) on cardiovascular events (CVE), progression to end-stage renal disease (ESRD) and all-cause mortality.

**Results:**

56.2% of individuals with CKD had NAFLD at baseline, and 3.0% and 7.7% had NAFLD fibrosis according to a FIB-4 > 2.67 and NFS ≥ 0.676, respectively. The median follow-up was 13 years. In univariate analysis, NAFLD was associated with an increased risk of CVE (HR 1.49 [1.38–1.60]), all-cause mortality (HR 1.22 [1.14–1.31]) and ESRD (HR 1.26 [1.02–1.54]). Following multivariable adjustment, NAFLD remained an independent risk factor for CVE overall (HR 1.20 [1.11–1.30], *p* < 0.0001), but not ACM or ESRD. In univariate analysis, elevated NFS and FIB-4 scores were associated with increased risk of CVE (HR 2.42 [2.09–2.80] and 1.64 [1.30–2.08]) and all-cause mortality (HR 2.82 [2.48–3.21] and 1.82 [1.47–2.24]); the NFS score was also associated with ESRD (HR 5.15 [3.52–7.52]). Following full adjustment, the NFS remained associated with an increased incidence of CVE (HR 1.19 [1.01–1.40]) and all-cause mortality (HR 1.31 [1.13–1.52]).

**Conclusions:**

In people with CKD, NAFLD is associated with an increased risk of CVE, and the NAFLD fibrosis score is associated with an elevated risk of CVE and worse survival.

**Supplementary Information:**

The online version contains supplementary material available at 10.1186/s12916-023-02891-x.

## Background

Non-alcoholic fatty liver disease (NAFLD) refers to the accumulation of excess fat in the liver and affects 25% of adults [1]. It can progress to non-alcoholic steatohepatitis (NASH), liver fibrosis, cirrhosis and hepatocellular carcinoma [2]. NAFLD is also an independent risk factor for cardiovascular disease (CVD) and death [3]. Indeed, CVD is the leading cause of mortality relating to NAFLD [4]. In addition, NAFLD is independently associated with an increased risk of chronic kidney disease (CKD) [5, 6]; this risk is particularly high where individuals have more advanced liver disease, i.e. NASH or hepatic fibrosis.

CKD is associated with reduced quality of life and increased risk of end-stage renal disease (ESRD), CVD and premature death [7, 8]. It carries a huge burden in terms of health care costs largely due to renal replacement therapy. ESRD is estimated to be associated with a mean annual health care cost of $20–100,000 per patient in developed countries [8]. The global prevalence of CKD stages 3–5 is approximately 11% [9], with the prevalence increasing by nearly a third since 2007 [10]. CKD is itself an accelerator of CVD risk and an independent risk factor for cardiovascular events (CVEs) [11–13]. As with NAFLD, the leading cause of death for patients with CKD is CVD [14].

The implications of having both NAFLD and CKD are poorly understood. We performed a systematic review examining the impact of NAFLD on clinical outcomes and mortality for people with CKD [15]. Only three studies were included, which were diverse in design with conflicting results. The first reported a positive association of NAFLD with all-cause mortality (ACM); however, significance was lost following adjustment for metabolic risk [16]; the second study reported no effect on mortality in unadjusted or adjusted models [17]. The third study observed NAFLD to be an independent risk factor for non-fatal CVE [17]. Two papers examined CKD progression; in one, the adjusted rate of decline in estimated glomerular filtration rate (eGFR) per year was higher in those with NAFLD [18], whereas the other found no significant difference [17]. Liver fibrosis, detected using non-invasive scores, was found to be associated with CKD progression [18] but not ACM [16]. Observational data from Japan has also shown an association between non-invasive markers of liver fibrosis and incident diabetic kidney disease [19].

NAFLD and CKD share cardio-metabolic risk factors but also pathophysiological mechanisms that can lead to end-stage disease (e.g. CVE and ESRD), including insulin resistance and the activation of pro-inflammatory and pro-fibrinogenic pathways. Understanding if NAFLD accelerates the development of adverse health outcomes and increases ACM for patients with CKD is highly clinically relevant. This will inform the need for risk stratification, enhanced lifestyle intervention, targeted pharmacological management of common risk factors and clinical trial enrolment. We therefore aimed to determine whether and to what extent NAFLD and NAFLD with advanced liver fibrosis are independently associated with the risk of CVE, progression to ESRD and ACM in people with CKD.

## Methods

### Study population

The UK Biobank (UKBB) is a national prospective cohort study aimed at improving disease prevention (http://www.ukbiobank.ac.uk/about-biobank-uk). Over 500,000 individuals aged 40–69 agreed to participate and were recruited between 2006 and 2010. During baseline assessment visits, participants completed questionnaires about their demographics, medical history and lifestyle. Self-reported doctor-diagnosed medical conditions were verified and coded during a face-to-face interview (https://biobank.ctsu.ox.ac.uk/crystal/field.cgi?id=20002). Volunteers underwent a physical examination and provided blood and urine samples. All participants gave consent to be followed up through linkage to electronic health records (death and cancer records held by the Office for National Statistics and the Registrar General’s Office; hospital records held by the Department of Health’s Hospital Episode Statistics; Scottish Morbidity Records). At the time of analysis, mortality and hospital admission data were available to January 2023. Ethical approval for the UKBB study was granted by the North West Multi-Centre Research Ethics Committee (06/MRE08/65).

### Inclusion criteria

We identified all participants within the UKBB who had evidence of CKD at their baseline visit determined by a single eGFR value (eGFR) < 60 ml/min/1.73 m^2^ or a random urine albumin creatinine ratio (UACR) ≥ 3 mg/mmol [20]. eGFR was calculated using single serum creatinine and cystatin C measurements, omitting race as per the most recent guidance [21]. We present the combined equation as this is a more valid measure [22] but undertake a sensitivity analysis using serum creatinine alone to calculate GFR [23], as is currently recommended for routine practice in UK [24]. Detailed information on how the urine samples were collected and the methods used to calculate the urine albumin creatinine ratio can be found in the supplementary material (Additional file 1: Supplementary methods).

### Exclusion criteria

Participants were excluded if they had evidence of baseline ESRD (eGFR < 15 ml/min/1.73 m^2^ or anyone identified to have ESRD according to the UKBB algorithm (data field 42,026) [25]. Participants were also excluded if they had undergone a liver transplant and had a non-NAFLD cause of liver disease at baseline (Additional file 2: Table S1), evidence of alcohol abuse (Additional file 3: Table S2) or a baseline alcohol intake of ≥ 20 g per day for women and ≥ 30 g per day for men. Approximately 27% of participants had data on the frequency of alcohol consumption only (i.e. not weekly grams); in this case, we excluded individuals drinking daily or more than daily. Finally, we excluded all participants who did not have data available to calculate the following scores at baseline: hepatic steatosis index (HSI), fibrosis-4 score (FIB-4) and NAFLD fibrosis score (NFS). These are validated algorithms comprising both clinical and biochemical parameters (calculations shown in Table 1).

**Table 1:** Algorithms used to calculate hepatic steatosis index and fibrosis scores

**Hepatitis Steatosis Index**	$8\times\left(\text{ALT}\left[\text{U/l}\right]/\text{AST}\left[\text{U/l}\right]\right)+\text{BMI}\left[\text{kg}/\mathrm{m}^2\right]\left(+2\,\text{if type 2 diabetes,}+2\,\text{if female}\right)$
**Fibrosis-4 index (FIB-4)**	$\text{Age}\left[\text{years}\right]\times\text{AST}\left[\text{U/l}\right]/\left(\text{platelets}\left[\times10^9/\mathrm{L}\right]\times\sqrt{}\left(\text{ALT}\left[\text{U/l}\right]\right)\right)$
**NAFLD fibrosis score (NFS)**	$-1.675+0.037\times\text{age}\left[\text{years}\right]+0.094\times\text{BMI}\left[\mathrm{kg}/\mathrm{m}^2\right]+1.13\times\left(\text{IFG or diabetes [yes = 1, no = 0]}\right)+0.99\times\text{AST}\left[\text{U/l}\right]/\text{ALT}\left[\text{U/l}\right]-0.013\times\text{platelets}\left[10^9/\mathrm{L}\right]-0.66\times\text{albumin}\left[\text{g/dl}\right]$

*ALT* alanine transaminase, *AST* aspartate transaminase, *BMI* body mass index, *IFG* impaired fasting glucose

### Ascertainment of exposure

Individuals were identified as having NAFLD if they had either an ICD-9 (571.8) or ICD-10 (K75.8, K76.0) code indicating NAFLD or an HSI > 36 [26]. Individuals were defined as not having NAFLD if they had no ICD code for NAFLD and HSI < 36. The influence of advanced fibrosis was examined in patients with CKD and NAFLD using the NFS [27, 28] and FIB-4 score[28, 29]. Advanced fibrosis was defined as NFS ≥ 0.676 or FIB-4 score > 2.67 at baseline. Advanced fibrosis was excluded if there was a NFS <  − 1.455 (< 0.12 if ≥ 65 years) or FIB-4 score < 1.3 (< 2.0 if ≥ 65 years) [16]. Participants not falling into these groups were placed in an indeterminate fibrosis group. We selected NFS and FIB-4 to identify participants with liver fibrosis because they are superior to other scores that predict the presence of liver fibrosis [28] and could both be calculated from the data collected in UKBB participants. The positive predictive value of NFS ≥ 0.676 and FIB-4 > 2.67 in predicting liver fibrosis in patients with NAFLD is 90% and 80%, respectively [28].

### Primary outcomes

Primary outcomes included the risk of incident CVE, progression to ESRD and ACM:

#### Cardiovascular events

A CVE was defined as an ICD code for a new diagnosis of any one of the following: acute coronary syndrome (ACS), heart failure (HF), cerebrovascular accident (CVA) (ischaemic or haemorrhagic stroke or transient ischemic event) or peripheral arterial disease (PAD) (Additional file 4: Table S3). This event could have been fatal or non-fatal. Where a participant may have experienced multiple CVE, the first was included. For the outcome of all CVE, all patients with evidence of prior CVE were excluded; for subgroups of CVE, just participants who reported that specific CVE at baseline were excluded (Additional file 4: Table S3).

#### Development of ESRD

Incident ESRD was defined using the UKBB ESRD algorithm. This was devised to identify participants who have had or are undergoing renal replacement therapy (RRT) using ICD-10 and OPCS-4 codes [25]. The algorithm selects only people with other diagnoses or procedures that indicate CKD stage 5, aiming to exclude those receiving RRT for acute kidney injury. Algorithmically derived ESRD results can be used to look at baseline ESRD and incident ESRD. The principles utilised by the algorithm have previously been used successfully [30].

#### All-cause mortality

ACM included any cause of death within the study follow-up period. The primary cause of death was gathered from the linked datasets described above.

### Statistical analysis

Baseline characteristics are presented as percentages, and continuous data are presented using the median and interquartile range (IQR). Univariate and multivariable Cox proportional hazards models were used to calculate the association of NAFLD and hepatic fibrosis (in people with NAFLD) on CVE, ESRD and ACM. Hazard ratios (HR) with 95% confidence intervals (CIs) are presented. Statistical significance was taken as *p* < 0.05. Non-cases were censored at the date of loss to follow-up, date of death or end of follow-up. Individuals were also censored for a CVE if they developed ESRD first, as it was thought ESRD would alter the course and mechanisms of cardiovascular injury and outweigh the influence of NAFLD. People were not censored for other subtypes of CVE having developed a different subtype. Multivariable adjustment was informed using direct acrylic graphs (DAGitty) [31]. Model 1 adjusted for age, sex, ethnicity and Townsend Deprivation Index (a place-based metric of socioeconomic status based on car ownership, home ownership, employment and over-crowding) [32]. Model 2 additionally adjusted for smoking status (never, previous, current), baseline eGFR (G1-5) and baseline UACR (A1-3) (Additional file 5: Table S4). Model 3 adjusted for the above factors in addition to diabetes (see Additional file 1: Supplementary methods for definitions). There was at least 80% power to detect a 15% increase in the hazard of all outcomes [33]. The linearity of the effect of each continuous variable in the adjusted models was determined using univariate Cox hazard regression with penalised splines. Where a Wald-type test using the nonlinear coefficient estimates indicated significant non-linearity, flexible splines were used in further analyses. The validity of the proportional hazards assumption for each variable was determined by examining correlations between scaled Schoenfeld residuals and time. The statistical package used was R. The Strengthening the Reporting of Observational Studies in Epidemiology (STROBE) guidelines were followed in reporting this study [34].

## Results

### Identification of a cohort of individuals with CKD within the UKBB

**Fig. 1:**
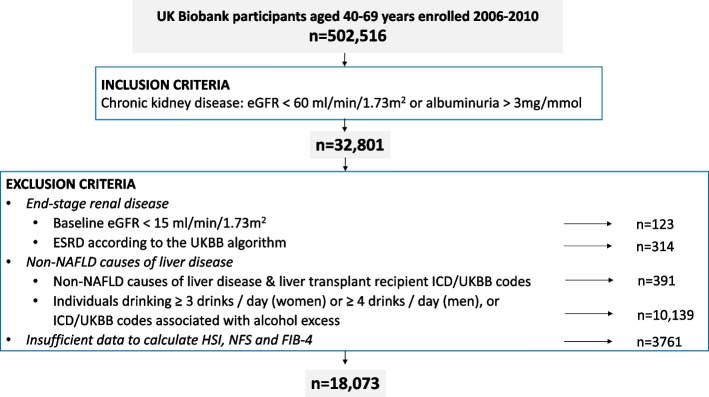
Flow chart of participant recruitment

Overall 455,260 UKBB participants had recorded baseline data for eGFR or albuminuria; 32,801 (7.2%) had evidence of CKD (Additional file 5: Table S4, Fig. 1). Following exclusions, the final study sample consisted of 18,073 participants with CKD (Fig. 1). For the analyses of CVE outcomes, a further 1458 people who had experienced a baseline CVE were excluded. Baseline demographics are presented in Table 2. The baseline characteristics of individuals excluded due to insufficient data to calculate the HSI and serum fibrosis scores did not differ significantly from those included (Additional file 6: Table S5).

**Table 2:** Comparison of baseline demographics between participants with and without NAFLD

	**Total**	**NAFLD status**		
		**No NAFLD**	**NAFLD**	**Standardised difference**
***N, (%)***	18,073	7921 (43.8)	10,152 (56.2)	
***Median age, years (IQR)***	62 (12)	62 (13)	62 (11)	0.048
***Male (%)***	42.1	39.6	44.1	0.093
***Ethnicity (%)***				0.039
White	89.4	90.0	88.9	
Non-white	10.0	9.5	10.5	
***Townsend deprivation index***				0.201
*Median score*	− 1.55	− 1.95	− 1.19	
***Alcohol***				
Weekly gram data available (%)	72.7	74.9	71.0	0.09
Mean alcohol grams per week	40.0	48.0	34.0	0.104
Non-drinkers (abstainers and former) (%)	17.4	15.8	18.7	
***Diabetes***				
Diabetes (%)	19.5	5.2	30.7	0.703
Median HbA1c people with diabetes, mmol/mol	54.7	52.9	55.0	0.15
Median HbA1c overall, mmol/mol	37.3	35.7	39.1	0.65
***Overweight/obesity***				
Median BMI, kg/m^2^ (IQR)	28.6 (7.7)	24.7 (3.9)	32.2 (6.2)	
Weight categories				2.09
Overweight (BMI 25–30 kg/m^2^) (%)	35.1	44.7	27.7	
Obese (BMI > 30 kg/m^2^) (%)	40.6	1.2	71.3	
Median waist circumference, cm (IQR)	95 (22)	83 (16)	103 (18)	
High risk (WC men > 102 cm, women > 88 cm) (%)	48.3	11.2	77.3	1.695
***Lipids***				
Dyslipidaemia (%)	72.0	55.4	84.9	0.68
Median HDL (mmol/L)	1.3	1.4	1.2	0.686
Median TG (mmol/L)	1.7	1.4	2.0	0.606
***Hypertensive (%)***	57.5	43.7	68.2	0.51
***Smoking (%)***				0.185
Never smoked	53.5	56.9	50.9	
Previous smoker	33.9	29.2	37.5	
Current smoker	11.6	13.1	10.5	
***Liver enzymes***				
Median ALT (IU/l)	21	17	25	0.646
Median AST (IU/l)	25	24	25	0.183
Median GGT (IU/l)	30	23	35	0.367
Median platelets (10^6^/l)	252	249	253	0.028
Median albumin (g/l)	45	45	45	0.129
***Baseline CVE (%)***	7.6	6.0	9.0	0.115
***Baseline eGFR***				
Median eGFR (ml/min/1.73 m^2^)	85	90	81	0.286
G1 (≥ 90 ml/min/1.73 m^2^) (%)	41.6	49.2	35.7	
G2 (60–89 ml/min/1.73 m^2^) (%)	29.9	27.6	31.7	
G3a (45–59 ml/min/1.73 m^2^) (%)	22.4	18.1	25.8	
G3b (30–44 ml/min/1.73 m^2^) (%)	4.8	3.9	5.4	
G4 (15–29 ml/min/1.73 m^2^) (%)	1.2	1.0	1.3	
***Baseline UACR***				
Median UACR (mg/mmol)	39	33	44	0.023
UACR < 3 mg/mmol (%)	20.7	16.8	23.8	
UACR 3–30 mg/mmol (%)	71.4	76.0	67.8	
UACR > 30 mg/mmol (%)	6.7	6.5	6.8	

*NAFLD* non-alcoholic fatty liver disease, *IQR* interquartile range, *HbA1c* glycated haemoglobin, *BMI* body mass index, *WC* waist circumference, *LDL* low-density lipoprotein cholesterol, *HDL* high-density lipoprotein cholesterol, *TG* triglycerides, *ALT* alanine transaminase, *AST* aspartate transaminase, *GGT* gamma glutamyl transferase, *eGFR* estimated glomerular filtration rate, *UACR* urine albumin creatinine ratio, *ns* not significant

### Prevalence of NAFLD and NAFLD fibrosis in the UKBB CKD cohort

In this CKD cohort, 56.2% (*n* = 10,152) of people were identified as having NAFLD. Those with NAFLD were more likely to be male and have a diagnosis of metabolic disease and lower baseline eGFR (Table 2). The prevalence of NAFLD risk-stratified according to CKD stage is presented in Additional file 7: Table S6. For people with CKD and NAFLD, 7.7% (*n* = 784) and 3.0% (*n* = 308) were identified as having advanced fibrosis according to NFS ≥ 0.676 or FIB-4 > 2.67, respectively. Individuals with advanced fibrosis were more likely to be male, have features of the metabolic syndrome, have experienced a prior CVE and have poorer baseline renal function (Additional file 8: Table S7).

### Outcomes: CVE, ESRD and all-cause mortality

The median follow-up time was 13.2 years for a CVE and 13.6 years for the development of ESRD and ACM. In total, 1666 individuals with baseline CKD developed a CVE, 215 progressed to ESRD and there were 1942 deaths. The event rates for incident CVE (fatal and non-fatal events), ESRD and ACM were higher for individuals with NAFLD (Additional file 9: Fig. S1). Univariate analysis of factors associated with increased HR of CVE, ESRD and ACM is shown in Additional file 10: Table S8. The event rates for all primary outcomes increased with increasing severity of CKD at baseline according to both eGFR and albuminuria (Additional file 11: Table S9).

### Association of NAFLD with CVE, ESRD and all-cause mortality

**Fig. 2:**
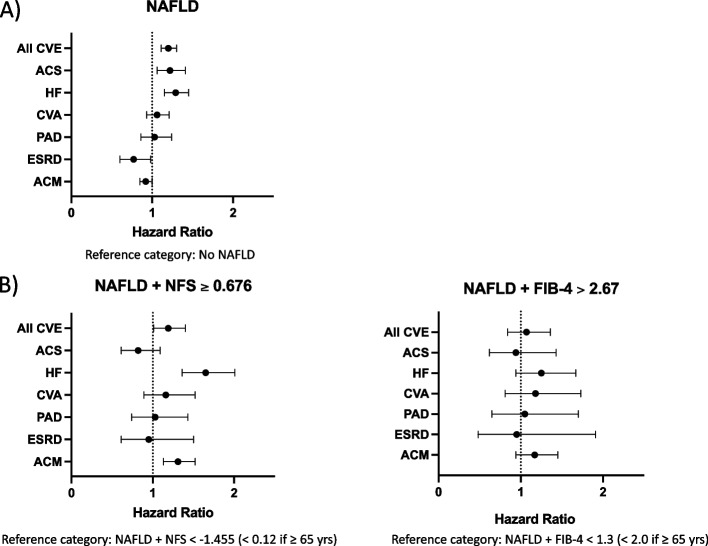
Hazard ratios and 95% confidence intervals for the association of **A** NAFLD and **B** advanced liver fibrosis in people with NAFLD, with primary outcomes for people with CKD following full multivariable adjustment

Univariate analysis revealed that NAFLD was associated with an increased risk of all CVE (HR 1.49 [1.38–1.60], *p* < 0.0001), ACM (HR 1.22 [1.14–1.31], *p* < 0.0001) and ESRD (HR 1.26 [1.02–1.54], *p* = 0.0298) (Table 3). Following multivariable adjustment for age, sex, ethnicity, deprivation, alcohol, smoking, baseline renal function and diabetes, NAFLD remained an independent risk factor for CVE overall (HR 1.20 [1.11–1.30], *p* < 0.0001), including ACS (HR 1.22 [1.06–1.41], *p* = 0.0057) and HF (HR 1.29 [1.15–1.45], *p* < 0.0001), but not ACM (HR 0.92 [0.85–1.00]) or ESRD (HR 0.77 [0.60–0.98]) (Table 3, Fig. 2). We examined the change in direction of the association between NAFLD and ACM after adjusting for diabetes, which is a component of HSI, by assessing for potential collinearity between NAFLD and diabetes. The Phi coefficient was 0.32, indicative of a moderate positive association. Although it is conceivable that collinearity played a role in the alteration of the association between NAFLD and ACM, the strength of collinearity was not sufficiently robust to draw definitive conclusions.

**Table 3:** Association of NAFLD with CVE, ESRD and all-cause mortality

		**Participants**	**Events**	**Median follow-up (months)**	**Event rate per person-year**	**Univariate model**	**Multivariable model 1**	**Multivariable model 2**	**Multivariable model 3**
**All cardiovascular events, HR (95% CI)**	No NAFLD	7921	587	160.8	0.012	1.00 ref	1.00 ref	1.00 ref	1.00 ref
	NAFLD	10,152	1079	157.6	0.018	1.49 (1.38–1.60)****	1.42 (1.32–1.53)****	1.40 (1.29–1.51)****	1.20 (1.11–1.30)****
**Acute coronary syndrome, HR (95% CI)**	No NAFLD	7921	191	163.2	0.004	1.00 ref	1.00 ref	1.00 ref	1.00 ref
	NAFLD	10,152	343	161.2	0.006	1.59 (1.40–1.82)****	1.52 (1.33–1.73)****	1.49 (1.30–1.70)****	1.22 (1.06–1.41)**
**Heart failure, HR (95% CI)**	No NAFLD	7921	274	163.5	0.005	1.00 ref	1.00 ref	1.00 ref	1.00 ref
	NAFLD	10,152	520	161.4	0.009	1.74 (1.57–1.93)****	1.67 (1.50–1.85)****	1.58 (1.41–1.75)****	1.29 (1.15–1.45)****
**Cerebrovascular accident, HR (95% CI)**	No NAFLD	7921	215	163.3	0.004	1.00 ref	1.00 ref	1.00 ref	1.00 ref
	NAFLD	10,152	422	161.8	0.006	1.31 (1.16–1.48)****	1.25 (1.11–1.42)***	1.25 (1.10–1.42)***	1.06 (0.93–1.21)
**Peripheral arterial disease, HR (95% CI)**	No NAFLD	7921	131	163.8	0.002	1.00 ref	1.00 ref	1.00 ref	1.00 ref
	NAFLD	10,152	227	162.6	0.003	1.48 (1.26–1.74)****	1.37 (1.16–1.61) ***	1.38 (1.17–1.64)***	1.03 (0.86–1.24)
**End-stage renal disease, HR (95% CI)**	No NAFLD	7921	67	164.0	0.001	1.00 ref	1.00 ref	1.00 ref	1.00 ref
	NAFLD	10,152	148	163.2	0.002	1.26 (1.02–1.54)*	1.16 (0.94–1.43)	1.10 (0.88–1.37)	0.77 (0.60–0.98)*
**All-cause mortality, HR (95% CI)**	No NAFLD	7921	688	164.3	0.012	1.00 ref	1.00 ref	1.00 ref	1.00 ref
	NAFLD	10,152	1254	163.4	0.015	1.22 (1.14–1.31)****	1.15 (1.07–1.24)***	1.09 (0.02–1.18)*	0.92 (0.85–1.00)*

Model 1: adjusted for age, sex, deprivation and ethnicity

Model 2: adjusted for age, sex, deprivation, ethnicity, smoking and baseline eGFR and UACR

Model 3: adjusted for age, sex, deprivation, ethnicity, smoking, baseline eGFR and UACR and diabetes

*NAFLD* non-alcoholic fatty liver disease, *HR* hazard ratio, *CI* confidence interval

^*^p < 0.05

^**^p < 0.01

^***^p < 0.001

^****^p < 0.0001

### Association of NAFLD fibrosis with CVE, ESRD and all-cause mortality

*NFS*: Univariate analysis revealed that the NFS was associated with ACM, elevated risk of CVE and ESRD (Table 4). Following multivariable adjustment for demographics, smoking, baseline renal function and diabetes, an NFS ≥ score 0.676 remained associated with increased risk of all CVE (HR 1.19 [1.01–1.40], *p* = 0.0424), HF (HR 1.65 [1.36–2.01], *p* < 0.0001) and ACM (HR 1.31 [1.13–1.52], *p* = 0.0005) (Table 5, Fig. 2).

**Table 4:** Association of the NAFLD fibrosis score on CVE, ESRD and all-cause mortality for individuals with CKD and NAFLD

		**Participants**	**Events**	**Median follow-up (months)**	**Event rate**	**Univariate model**	**Multivariable model 1**	**Multivariable model 2**	**Multivariable model 3**
**All cardiovascular events, HR (95% CI)**	Low risk	6057	1835	161.0	0.013	1.00 ref	1.00 ref	1.00 ref	1.00 ref
	Intermediate risk	3311	905	155.7	0.02	1.46 (1.33–1.61)****	1.49 (1.35–1.65)****	1.40 (1.27–1.55)****	1.22 (1.10–1.36)***
	High risk	784	249	120.5	0.039	2.42 (2.09–2.80)****	1.84 (1.59–2.14)****	1.56 (1.33–1.82)****	1.19 (1.01–1.40)*
**Acute coronary syndrome, HR (95% CI)**	Low risk	6057	621	163.6	0.004	1.00 ref	1.00 ref	1.00 ref	1.00 ref
	Intermediate risk	3311	319	159.5	0.006	1.51 (1.28–1.77)****	1.46 (1.24–1.72)****	1.35 (1.14–1.60)***	1.08 (0.90–1.29)
	High risk	784	80	152.7	0.01	1.93 (1.50–2.50)****	1.44 (1.11–1.87)**	1.21 (0.92–1.59)	0.82 (0.61–1.09)
**Heart failure, HR (95% CI)**	Low risk	6057	908	163.9	0.006	1.00 ref	1.00 ref	1.00 ref	1.00 ref
	Intermediate risk	3311	528	159.6	0.01	1.63 (1.43–1.85)****	1.74 (1.52–1.99)****	1.63 (1.43–1.87)****	1.40 (1.21–1.61)****
	High risk	784	202	151.5	0.024	3.48 (2.93–4.12)****	2.68 (2.25–3.19)****	2.21 (1.84–2.65)****	1.65 (1.36–2.01)****
**Cerebrovascular accident, HR (95% CI)**	Low risk	6057	696	163.9	0.004	1.00 ref	1.00 ref	1.00 ref	1.00 ref
	Intermediate risk	3311	323	159.8	0.006	1.28 (1.09–1.51)**	1.32 (1.12–1.56)**	1.28 (1.08–1.52)**	1.07 (0.89–1.28)
	High risk	784	94	153.4	0.01	2.18 (1.72–2.75)****	1.70 (1.34–2.16)****	1.60 (1.25–2.06)***	1.16 (0.89–1.52)
**Peripheral arterial disease, HR (95% CI)**	Low risk	6057	370	164.4	0.002	1.00 ref	1.00 ref	1.00 ref	1.00 ref
	Intermediate risk	3311	212	160.6	0.004	1.60 (1.30–1.96)****	1.61 (1.31–1.99)****	1.46 (1.17–1.81)***	1.12 (0.89–1.41)
	High risk	784	68	154.8	0.007	2.74 (2.05–3.66)****	2.00 (1.49–2.69)****	1.59 (1.16–2.16)**	1.03 (0.74–1.43)
**End-stage renal disease, HR (95% CI)**	Low risk	6057	160	164.8	0.001	1.00 ref	1.00 ref	1.00 ref	1.00 ref
	Intermediate risk	3311	176	161.2	0.003	3.08 (2.30–4.11)****	2.78 (2.07–3.72)****	1.68 (1.23–2.30)**	1.28 (0.92–1.77)
	High risk	784	48	155.5	0.005	5.15 (3.52–7.52)****	4.37 (2.96–6.46)****	1.55 (1.01–2.36)*	0.95 (0.61–1.50)
**All-cause mortality, HR (95% CI)**	Low risk	6057	1952	165.0	0.012	1.00 ref	1.00 ref	1.00 ref	1.00 ref
	Intermediate risk	3311	901	161.7	0.016	1.34 (1.22–1.48)****	1.44 (1.30–1.59)****	1.31 (1.18–1.45)****	1.12 (1.00–1.25)*
	High risk	784	342	156.2	0.034	2.82 (2.48–3.21)****	2.13 (1.87–2.43)****	1.73 (1.50–1.99)****	1.31 (1.13–1.52)***

Model 1: adjusted for age, sex, deprivation and ethnicity

Model 2: adjusted for age, sex, deprivation, ethnicity, smoking and baseline eGFR and UACR

Model 3: adjusted for age, sex, deprivation, ethnicity, smoking, baseline eGFR and UACR and diabetes

Low risk fibrosis: NAFLD fibrosis score <  − 1.455 (< 0.12 if ≥ 65 years)

Intermediate risk fibrosis: NAFLD fibrosis score − 1.455–0.676 (0.12–0.676 if ≥ 65 years)

High-risk fibrosis: NAFLD fibrosis score ≥ 0.676

*HR* hazard ratio, *CI* confidence interval

^*^p < 0.05

^**^p < 0.01

^***^p < 0.001

^****^p < 0.0001

**Table 5:** Association of the Fibrosis-4 score on CVE, ESRD and all-cause mortality for individuals with CKD and NAFLD

		**Participants**	**Events**	**Median follow-up (months)**	**Event rate**	**Univariate model**	**Multivariable model 1**	**Multivariable model 2**	**Multivariable model 3**
**All cardiovascular events, HR (95% CI)**	Low risk	7202	1887	160.4	0.014	1.00 ref	1.00 ref	1.00 ref	1.00 ref
	Intermediate risk	2642	949	156.9	0.017	1.28 (1.16–1.41)****	1.14 (1.03–1.26)*	1.11 (1.00–1.23)*	1.13 (1.02–1.25)*
	High risk	308	153	145.3	0.027	1.64 (1.30–2.08)****	1.17 (0.92–1.48)	1.09 (0.85–1.39)	1.07 (0.84–1.36)
**Acute coronary syndrome, HR (95% CI)**	Low risk	7202	659	163.4	0.005	1.00 ref	1.00 ref	1.00 ref	1.00 ref
	Intermediate risk	2642	314	160.1	0.005	1.20 (1.01–1.42)*	1.05 (0.88–1.24)	1.01 (0.85–1.20)	1.03 (0.86–1.22)
	High risk	308	47	154.3	0.007	1.36 (0.90–2.05)	0.95 (0.63–1.45)	0.941.46 (0.6293–1.442.30)	0.94 (0.62–1.43)
**Heart failure, HR (95% CI)**	Low risk	7202	1025	163.5	0.007	1.00 ref	1.00 ref	1.00 ref	1.00 ref
	Intermediate risk	2642	512	160.6	0.008	1.25 (1.09–1.42)***	1.12 (0.98–1.28)	1.08 (0.95–1.24)	1.11 (0.97–1.27)
	High risk	308	101	153.8	0.014	1.86 (1.39–2.47)****	1.32 (0.99–1.75)	1.25 (0.93–1.66)	1.25 (0.94–1.67)
**Cerebrovascular accident, HR (95% CI)**	Low risk	7202	683	163.6	0.005	1.00 ref	1.00 ref	1.00 ref	1.00 ref
	Intermediate risk	2642	367	160.5	0.006	1.18 (1.00–1.39)	1.07 (0.90–1.26)	1.06 (0.89–1.26)	1.08 (0.91–1.28)
	High risk	308	63	154.6	0.008	1.77 (1.24–2.53)**	1.26 (0.87–1.82)	1.19 (0.81–1.74)	1.18 (0.81–1.73)
**Peripheral arterial disease, HR (95% CI)**	Low risk	7202	414	164.2	0.003	1.00 ref	1.00 ref	1.00 ref	1.00 ref
	Intermediate risk	2642	195	161.4	0.003	1.24 (1.00–1.53)*	1.08 (0.87–1.33)	1.06 (0.85–1.31)	1.08 (0.87–1.35)
	High risk	308	41	155.8	0.005	1.61 (1.00–2.59)	1.11 (0.68–1.79)	1.01 (0.62–1.64)	1.05 (0.65–1.70)
**End-stage renal disease, HR (95% CI)**	Low risk	7202	225	164.5	0.001	1.00 ref	1.00 ref	1.00 ref	1.00 ref
	Intermediate risk	2642	139	156.9	0.002	1.39 (1.06–1.83)*	1.25 (0.94–1.65)	0.94 (0.70–1.28)	0.97 (0.72–1.31)
	High risk	308	20	145.3	0.002	1.51 (0.77–2.96)	1.31 (0.67–2.59)	0.73 (0.36–1.46)	0.95 (0.48–1.91)
**All-cause mortality, HR (95% CI)**	Low risk	7202	2033	164.7	0.013	1.00 ref	1.00 ref	1.00 ref	1.00 ref
	Intermediate risk	2642	935	156.9	0.013	1.10 (0.99–1.21)	1.00 (0.90–1.10)	0.94 (0.85–1.05)	0.96 (0.87–1.07)
	High risk	308	227	145.3	0.028	1.82 (1.47–2.24)****	1.27 (1.03–1.57)****	1.16 (0.93–1.44)	1.17 (0.94–1.45)

Model 1: adjusted for age, sex, deprivation and ethnicity

Model 2: adjusted for age, sex, deprivation, ethnicity, smoking and baseline eGFR and UACR

Model 3: adjusted for age, sex, deprivation, ethnicity, smoking, baseline eGFR and UACR and diabetes

Low-risk fibrosis: Fibrosis-4 score < 1.3 (< 2.0 if ≥ 65 years)

Intermediate risk fibrosis: Fibrosis-4 score 1.3–2.67 (2.0–2.67 if ≥ 65 years)

High-risk fibrosis: Fibrosis-4 score > 2.67

*HR* hazard ratio, *CI* confidence interval

^*^p < 0.05

^**^p < 0.01

^***^p < 0.001

^****^p < 0.0001

*FIB-4 score*: A high FIB-4 score was associated with ACM and all CVE including HF and CVA in univariate analysis, but these associations lost statistical significance following full multivariable adjustment (Table 4, Fig. 2).

### Sensitivity analyses

In a sensitivity analysis in which eGFR was defined using creatinine alone, the NAFLD fibrosis score was no longer associated with an increased risk of CVE following multivariable adjustment; however, the findings were otherwise comparable (Additional file 12: Table S10). A further sensitivity analysis was performed where CKD was defined according to eGFR < 60 ml/min/1.73 m^2^ alone, albuminuria ≥ 3 mg/mmol alone, or both (Additional file 13: Table S11). In all analyses, NAFLD remained associated with an increased incidence of CVE.

## Discussion

The prevalence of NAFLD is this CKD cohort is 56%. NAFLD is significantly associated with CVE, ACM and ESRD in univariate analysis and remained associated with elevated CVE incidence following full adjustment for covariates in people with CKD. Prevalence rates of advanced fibrosis are estimated to be 3.0–7.7% for people with CKD and NAFLD. In this setting, a raised NFS was independently associated with ACM and CVE, in particular, heart failure. While an elevated FIB-4 score demonstrated a similar trend for both these outcomes it failed to reach statistical significance.

Results from this study validate findings that NAFLD overall is not associated with increased ACM or ESRD following multivariable adjustment for people with CKD [16, 17]. In common with our results, the UK Salford group reported that NAFLD was associated with non-fatal CVE in a propensity-matched group [17]. A key finding of this paper is the influence of the NAFLD fibrosis score on CVE and ACM for people with CKD. While a high FIB-4 score demonstrated a similar direction for both outcomes, it failed to reach statistical significance perhaps due to lower numbers of included participants (*n* = 308) compared to the NFS score (*n* = 784). The differences seen may also be due to the fact that the NFS score identifies a cohort of patients with more metabolic disease which may be mechanistically significant. Liver fibrosis is predictive of the risk of end-stage liver events [35–40]. Non-invasive markers of liver fibrosis (designed to avoid liver biopsy) can also predict hepatic decompensation and liver-related deaths [41–46], in addition to non-liver-related events. Results from the third NHANES study show that NFS and FIB-4 are associated with increased ACM and death from CVD [47]. Large prospective studies have also shown NFS and FIB-4 to be independent predictors of CVE [48, 49]. In the CKD population, the NHANES dataset showed that fibrosis was not significantly associated with all-cause or cardiovascular-related mortality; overall, numbers were low however (*n* = 60) [16]. Data from South Korea showed a raised NFS ≥  − 1.455 to be associated with greater deterioration in eGFR in patients with CKD [18]. While we demonstrate a significant association between an NFS score ≥ 0.676 and ESRD in model 2, this relationship is lost following adjustment for diabetes status.

We found that raised serum fibrosis markers are strongly associated with heart failure. NAFLD has been linked to left ventricular diastolic dysfunction [50], heart failure with preserved ejection fraction [51], cardiomyopathy and arrhythmias [52]. Proposed mechanisms of injury include endothelial dysfunction, expansion of epicardial adipose tissue, coronary microcirculatory dysfunction, cardiac hypertrophy and myocardial fibrosis [53]. A small percentage may have developed cirrhotic cardiomyopathy. These pathophysiological changes manifest in CKD too, so there may be an interaction between NAFLD fibrosis and CKD which increases heart failure risk. As far as we are aware, we are the first group to demonstrate a relationship between NAFLD fibrosis with heart failure prospectively and the first to examine this in the context of CKD.

This is the largest cohort of CKD patients in which the impact of having NAFLD on multimorbid clinical outcomes and ACM has been examined and is the first prospective study in this field. Consequently, we were able to examine the influence of NAFLD and NAFLD fibrosis and identify that this is a key determinant of adverse clinical outcomes in this cohort. The UKBB benefits from a robust methodology for baseline assessment and patients identified to have CKD were drawn from the general population so are more representative of people with CKD overall. Follow-up rates are high, as a result of linked routine data.

UKBB participants did not however undergo baseline ultrasound to look for hepatic steatosis; thus, our definition of NAFLD was predominantly based on the HSI. This score is endorsed for population screening of NAFLD [26]; however, there is limited evidence indicating how it performs in patients with CKD. A single study, consisting of two cohorts of patients with CKD, showed that the HSI was significantly associated with steatosis on liver ultrasound in one but not the other [54]. If this limitation introduced bias into our study, it would have reduced the significance of the associations we observed. The low number of people with NAFLD defined using ICD codes (*n* = 63) precluded any meaningful analysis of this group on its own. This number is low as most patients with NAFLD are managed in the community, or an outpatient setting or have undetected disease.

Neither the FIB-4 nor NFS score has been specifically validated in patients with CKD (although patients with CKD were not excluded from validation studies) [28]. This is potentially important as conceivably scores might be elevated due to fibrotic processes occurring in other organs including the kidneys and heart and different scores might be affected differently by these pathophysiological processes. Patented serum fibrosis markers and transient elastography may help clarify the association of liver fibrosis with clinical outcomes and improve the predictive value of non-invasive scores in this setting, but unfortunately, this data is not available in the UKBB. We had to base the diagnosis of CKD on a single measurement of eGFR or albuminuria, so we could not verify that selected participants had persistent changes consistent with a diagnosis of CKD for at least 3 months [20]. This may have resulted in a small number of false-positive diagnoses of CKD. Furthermore, our definition of CKD progression was limited to the development of ESRD as data on repeated eGFR measurements was not available. The majority of participants had early CKD at baseline, and follow-up was just over 10 years which may have been too short to capture all eventual progression to ESRD. Detection of ESRD may also have been confounded by the fact that a significant proportion of participants could have died prior to the development of ESRD.

The clinical consequences of having NAFLD for people with CKD were previously unclear [15]. Our findings suggest that in individuals with CKD, assessment for NAFLD and NAFLD fibrosis may guide risk stratification for end-organ complications. It is envisaged this group would benefit from more stringent control of cardiometabolic risk factors via lifestyle and pharmacological interventions. Any delay or prevention of the development of a CVE would lead to significant improvements in quality of life and substantial cost savings for the health service. While the adjusted HRs associated with having NAFLD and NAFLD fibrosis are modest, they are likely to be higher in targeted groups within the greatest cardiometabolic risk. It is beyond the remit of this paper to assess the predictive value of assessing liver fibrosis on top of existing risk stratification tools for patients with CKD and therefore the effectiveness and cost-effectiveness of screening for NAFLD and fibrosis in this cohort; however, many individuals with CKD will have metabolic risk factors which should prompt consideration of an ultrasound for NAFLD [55, 56].

## Conclusions

These findings highlight an important relationship between the kidneys and the liver that is under-researched. Further exploration of the mechanisms behind the observed association between liver steatosis and fibrosis and all-cause mortality and cardiovascular outcomes in patients with CKD is warranted. Our results have implications for enhanced recognition of the co-existence of NAFLD and NAFLD fibrosis in patients with CKD and inform the need for further work to examine the predictive power of more robust measures of liver fibrosis on major clinical events in this group.

## Supplementary Information

**Additional file 1.** Supplementary methods.

**Additional file 2: Table S1.** List of ICD-9, ICD-10 and self-reported AQUKBB codes to exclude participants with evidence of a non-NAFLD cause of liver disease at baseline.

**Additional file 3: Table S2.** List of ICD-9, ICD-10 and self-reported UKBB codes to exclude participants with evidence of alcohol abuse.

**Additional file 4: Table S3.** List of ICD-9 and ICD-10 codes used to define a cardiovascular event outcome and for the exclusion of a baseline cardiovascular event.

**Additional file 5: Table S4.** Number and proportion of patients in each Kidney Disease: Improving Global Outcomecategory according to baseline albuminuria and eGFR results.

**Additional file 6: Table S5.** Comparison of baseline demographics between participants excluded due to no data available to calculate HSI, FIB-4 and NFS and study cohort.

**Additional file 7: Table S6.** Number and proportion of patients with NAFLD identified at baseline in each Kidney Disease: Improving Global Outcomecategory according to baseline albuminuria and eGFR results.

**Additional file 8: Table S7.** Comparison of baseline demographics between participants with and without NAFLD fibrosis.

**Additional file 9: Figure S1.** Primary outcome event rates for individuals with and without NAFLD.

**Additional file 10: Table S8.** Univariate analysis of factors predictive of primary outcomes for people with CKD.

**Additional file 11: Table S9.** Event ratesof primary outcome events according to baseline Kidney Disease: Improving Global Outcomecategory.

**Additional file 12: Table S10.** Sensitivity analysis showing the association of NAFLD with CVEs, ESRD and all-cause mortality where eGFR was calculated using serum creatinine alone.

**Additional file 13: Table S11.** Sensitivity analysis showing the association of NAFLD with CVEs, ESRD and all-cause mortality where CKD is defined according toeGFR < 60 ml/min/1.73m2 alone,albuminuria > 3mg/mmol alone, andfor patients meeting both criteria.

## Data Availability

Data used for this project can be accessed by contacting the UK Biobank.

## Abbreviations

ACM: All-cause mortality
ACS: Acute coronary syndrome
CI: Confidence interval
CKD: Chronic kidney disease
CVA: Cerebrovascular accident
CVD: Cardiovascular disease
CVE: Cardiovascular event
eGFR: Estimated glomerular filtration rate
ESRD: End-stage renal disease
FIB-4: Fibrosis-4 score
HF: Heart failure
HR: Hazard ratio
HSI: Hepatic steatosis index
ICD: International Classification of Disease
IQR: Interquartile range
NAFLD: Non-alcoholic fatty liver disease
NASH: Non-alcoholic steatohepatitis
NFS: Non-alcoholic fatty liver disease fibrosis score
PAD: Peripheral arterial disease
RRT: Renal replacement therapy
STROBE: Strengthening the Reporting of Observational Studies in Epidemiology
UACR: Urine albumin creatinine ratio
UKBB: United Kingdom Biobank
