# Supplementary material for: The impact of non-alcoholic fatty liver disease and liver fibrosis on adverse clinical outcomes and mortality in patients with chronic kidney disease: a prospective cohort study using the UK Biobank
Source: BMC Med. 2023 May 18;21:185. doi: 10.1186/s12916-023-02891-x (PMC10193672; doi:10.1186/s12916-023-02891-x)
Supplement: Supplementary file 1 — Additional file 1. Supplementary methods. [file 12916_2023_2891_MOESM1_ESM.docx]

**Supplementary Material**

**Methods**

*Collection of urine* (UK Biobank: Protocol for a large-scale prospective epidemiological resource; <https://www.ukbiobank.ac.uk/media/gnkeyh2q/study-rationale.pdf>). UKBB participants were asked to provide a urine sample and were given a urine collection pot and bar-coded vacutainer. Urine from the urine collection vessel was transferred to the pre-assigned bar-coded vacutainers. All vacutainers were maintained at 4^o^C until ready for packing and dispatch to the coordinating centre laboratory in temperature-controlled shipping boxes. When the vacutainers arrived at the central laboratory they were processed as soon as possible. All the vacutainers that arrived were scanned and compared against the data file from the assessment centres. The urine vacutainers were transferred to a customised TECAN liquid handling platform configured to maintain the samples at 4^o^C. We have now added this data to the supplementary material.

*Methods to measure urinary albumin*: Albumin was measured in the UK Biobank samples using the immuno-turbidimetric analysis method (Randox Biosciences, UK) while creatinine was measured using the enzymatic analysis method (Beckman Coulter, UK). If albumin was <6.7 mg/L (the assay detection level in UK Biobank) then albumin was set at 6.7 mg/L prior to the calculation of the ratio.

*Calculated of urinary albumin creatinine ratio (UACR)*: We assumed a urine albumin of 3.35 mg/L in people with an undetectable measurement (i.e. "<6.7mg/L"). We used 3.35 mg/L in this instance as it is the midpoint of 0-6.7 mg/L). Of the 5,999 participants with a urine albumin of “<6.7mg/L", 4,916, 1,073 and 10, respectively, ended up in the A1, A2 and A3 categories. Similarly, we assumed a urine creatinine of 44 micromole/L in people with an undetectable reading of "<88 micromole/L". This only affected 4 participants. We calculated UACR in mg/mmol = urine albumin (mg/L) / urine creatinine (micromole/L) / 1000. We checked a random selection of calculated UACRs against an online calculator (<https://www.mdcalc.com/urinary-protein-excretion-estimation>). The online calculator estimates UACR in mg/g, so we used another website for the conversion <http://www.scymed.com/en/smnxps/psdjb222_c.htm>. We used the following categories for A1-3: A1 = 0-<3 mg/mmol; A2 = ≥3-<30 mg/mmol; A3 = ≥30 mg/mmol. These were ordered such that A1 = 0-<3 mg/mmol was the reference category.

*Definition of confounders*

Diabetes: a history of physician-diagnosed diabetes recorded by the participant, or coded for in prior hospital records, use of antidiabetic drugs, or a random glucose ≥ 11.1 mmol/L mg/dl or HbA1c ≥ 48mmol/mol)

Hypertension: a history of hypertension recorded by the participant or coded for in hospital records, or being on antihypertensive therapy

Dyslipidaemia: a history of high cholesterol recorded by the participant, the use of lipid-lowering medication, or high density lipoprotein cholesterol (HDL) < 40 mg/dl men, < 50 mg/dl women, or triglycerides ≥ 150 mg/dl).
