# Supplementary material for: The impact of non-alcoholic fatty liver disease and liver fibrosis on adverse clinical outcomes and mortality in patients with chronic kidney disease: a prospective cohort study using the UK Biobank
Source: BMC Med. 2023 May 18;21:185. doi: 10.1186/s12916-023-02891-x (PMC10193672; doi:10.1186/s12916-023-02891-x)
Supplement: Supplementary file 2 — Additional file 2: Table S1. List of ICD-9, ICD-10 and self-reported AQUKBB codes to exclude participants with evidence of a non-NAFLD cause of liver disease at baseline. [file 12916_2023_2891_MOESM2_ESM.docx]

**Supplementary Table 1** List of ICD-9, ICD-10 and self-reported UKBB codes to exclude participants with evidence of a non-NAFLD cause of liver disease at baseline

| **UKBB code** | **Code type** | **Code** |
| --- | --- | --- |
| 1604 | UKBB SR | 1604 Alcoholic liver disease / alcoholic cirrhosis |
| 1156 | UKBB SR | 1156 Infective/viral hepatitis |
| 1159 | UKBB SR | 1159 Bile duct disease |
| 1160 | UKBB SR | 1160 Cholangitis |
| 1579 | UKBB SR | 1579 Hepatitis B |
| 1580 | UKBB SR | 1580 Hepatitis C |
| 1581 | UKBB SR | 1581 Hepatitis D |
| 1582 | UKBB SR | 1582 Hepatitis E |
| 1506 | UKBB SR | 1506 Primary biliary cirrhosis |
| 1507 | UKBB SR | 1507 Haemochromatosis |
| 202 | UKBB SR | 202 Liver/hepatocellular cancer |
| NA | ICD9 | 070 Viral Hepatitis |
| 700 | ICD9 | 070.0 Viral hepatitis A with hepatic coma |
| 701 | ICD9 | 070.1 Viral hepatitis A without mention of hepatic coma |
| 702 | ICD9 | 070.2 Viral hepatitis B with hepatic coma |
| 703 | ICD9 | 070.3 Viral hepatitis B without mention of hepatic coma |
| 704 | ICD9 | 070.4 Other specified viral hepatitis with hepatic coma |
| 705 | ICD9 | 070.5 Other specified viral hepatitis without mention of hepatic coma |
| 706 | ICD9 | 070.6 Unspecified viral hepatitis with hepatic coma |
| 709 | ICD9 | 070.9 Unspecified viral hepatitis without mention of hepatic coma |
| 1550 | ICD9 | 155.0 Malignant neoplasm of liver primary |
| 2751 | ICD9 | 275.1 Wilson's disease |
| 27502 | ICD9 | 275.02 Hemochromatosis |
| 27761 | ICD9 | 277.61 Alpha-1-antitrypsin deficiency |
| 4530 | ICD9 | 453.0 Budd Chiari |
| NA | ICD9 | 571 Alcoholic fatty liver disease |
| 5710 | ICD9 | 571.0 Alcoholic fatty liver disease |
| 5711 | ICD9 | 571.1 Acute alcoholic hepatitis |
| 5712 | ICD9 | 571.2 Alcoholic cirrhosis of liver |
| 5713 | ICD9 | 571.3 Alcoholic liver damage unspecified |
| 5714 | ICD9 | 571.4 Chronic hepatitis unspecified (includes autoimmune hepatitis) |
| 5716 | ICD9 | 571.6 Biliary cirrhosis |
| 5761 | ICD9 | 576.1 Cholangitis |
| NA | ICD10 | K70 Alcoholic liver disease |
| K700 | ICD10 | K70.0 Alcoholic fatty liver |
| K701 | ICD10 | K70.1 Alcoholic fatty liver |
| K702 | ICD10 | K70.2 Alcoholic fibrosis and sclerosis of liver |
| K703 | ICD10 | K70.3 Alcoholic cirrhosis of liver |
| K704 | ICD10 | K70.4 Alcoholic hepatic failure |
| K709 | ICD10 | K70.9 Alcoholic liver disease unspecified |
| K852 | ICD10 | K85.2 Alcohol-induced acute pancreatitis |
| K860 | ICD10 | K86.0 Alcohol-induced chronic pancreatitis |
| NA | ICD10 | B16 Acute hepatitis B |
| B160 | ICD10 | B16.0 Acute hepatitis B with delta-agent with hepatic coma |
| B161 | ICD10 | B16.1 Acute hepatitis B with delta-agent without hepatic coma |
| B162 | ICD10 | B16.2 Acute hepatitis B without delta-agent with hepatic coma |
| B169 | ICD10 | B16.9 Acute hepatitis B without delta-agent and without hepatic coma |
| NA | ICD10 | B17 Other acute viral hepatitis |
| B170 | ICD10 | B17.0 Acute delta-(super) infection of hepatitis B carrier |
| B171 | ICD10 | B17.1 Acute hepatitis C |
| B172 | ICD10 | B17.2 Acute hepatitis E |
| B178 | ICD10 | B17.8 Other specified acute viral hepatitis |
| B179 | ICD10 | B17.9 Acute viral hepatitis, unspecified |
| NA | ICD10 | B18 Chronic viral hepatitis |
| B180 | ICD10 | B18.0 Chronic viral hepatitis B with delta-agent |
| B181 | ICD10 | B18.1 Chronic viral hepatitis B without delta-agent |
| B182 | ICD10 | B18.2 Chronic viral hepatitis C |
| B188 | ICD10 | B18.8 Other chronic viral hepatitis |
| B189 | ICD10 | B18.9 Chronic viral hepatitis unspecified |
| NA | ICD10 | B19 Unspecified viral hepatitis |
| B190 | ICD10 | B19.0 Unspecified viral hepatitis with hepatic coma |
| NA | ICD10 | B19.1 Unspecified viral hepatitis B |
| NA | ICD10 | B19.2 Unspecified viral hepatitis C |
| B199 | ICD10 | B19.9 Unspecified viral hepatitis without hepatic coma |
| C220 | ICD10 | C22.0 Liver cell carcinoma |
| E830 | ICD10 | E83.0 Wilson's disease |
| E831 | ICD10 | E83.1 Hemochromatosis |
| E880 | ICD10 | E88.0 Alpha-1-antitrypsin deficiency |
| I820 | ICD10 | I82.0 Budd Chiari |
| NA | ICD10 | K71 Toxic liver disease |
| K710 | ICD10 | K71.0 Toxic liver disease with cholestasis |
| K711 | ICD10 | K71.1 Toxic liver disease with hepatic necrosis |
| K712 | ICD10 | K71.2 Toxic liver disease with acute hepatitis |
| K713 | ICD10 | K71.3 Toxic liver disease with chronic persistent hepatitis |
| K714 | ICD10 | K71.4 Toxic liver disease with chronic lobular hepatitis |
| K715 | ICD10 | K71.5 Toxic liver disease with chronic active hepatitis |
| K716 | ICD10 | K71.6 Toxic liver disease with hepatitis not elsewhere classified |
| K717 | ICD10 | K71.7 Toxic liver disease with fibrosis and cirrhosis of liver |
| K718 | ICD10 | K71.8 Toxic liver disease with other disorders of liver |
| K719 | ICD10 | K71.9 Toxic liver disease unspecified |
| K732 | ICD10 | K73.2 Chronic active hepatitis, not elsewhere classified |
| K739 | ICD10 | K73.9 Chronic hepatitis, unspecified |
| K743 | ICD10 | K74.3 Primary biliary cirrhosis |
| K744 | ICD10 | K74.4 Secondary biliary cirrhosis |
| K745 | ICD10 | K74.5 Biliary cirrhosis, unspecified |
| K754 | ICD10 | K75.4 Autoimmune hepatitis |
| K765 | ICD10 | K76.5 Hepatic veno-occlusive disease |
| K830 | ICD10 | K83.0 Primary sclerosing cholangitis |
