# Supplementary material for: The impact of non-alcoholic fatty liver disease and liver fibrosis on adverse clinical outcomes and mortality in patients with chronic kidney disease: a prospective cohort study using the UK Biobank
Source: BMC Med. 2023 May 18;21:185. doi: 10.1186/s12916-023-02891-x (PMC10193672; doi:10.1186/s12916-023-02891-x)
Supplement: Supplementary file 3 — Additional file 3: Table S2. List of ICD-9, ICD-10 and self-reported UKBB codes to exclude participants with evidence of alcohol abuse. [file 12916_2023_2891_MOESM3_ESM.docx]

**Supplementary Table 2** List of ICD-9, ICD-10 and self-reported UKBB codes to exclude participants with evidence of alcohol abuse

| **UKBB code** | **Code type** | **Code** |
| --- | --- | --- |
| 1408 | UKBB SR | 1408 Alcohol dependency |
| NA | ICD9 | 291 Alcoholic psychoses |
| 2910 | ICD9 | 291.0 Delerium tremens |
| 2911 | ICD9 | 291.1 Korsakov's psychosis alcoholic |
| 2912 | ICD9 | 291.2 Other alcoholic dementia |
| 2913 | ICD9 | 291.3 Other alcoholic hallucinations |
| 2914 | ICD9 | 291.4 Pathological drunkenness |
| 2915 | ICD9 | 291.5 Alcoholic jealousy |
| 2918 | ICD9 | 291.8 Other specified alcoholic psychoses |
| 2919 | ICD9 | 291.9 Alcoholic psychoses unspecified |
| NA | ICD9 | 303 Alcohol dependence syndrome |
| 3039 | ICD9 | 303.9 Other and unspecified alcohol dependence |
| 3050 | ICD9 | 305.0 Nondependent alcohol abuse |
| 3575 | ICD9 | 357.5 Alcoholic polyneuropathy |
| 4255 | ICD9 | 425.5 Alcoholic cardiomyopathy |
| 5353 | ICD9 | 535.3 Alcoholic gastritis |
| 9800 | ICD9 | 980.1 Toxic effect of ethyl alcohol |
| 9809 | ICD9 | 980.9 Toxic effect of unspecified alcohol |
| E244 | ICD10 | E24.4 Alcohol-induced pseudo-Cushing's syndrome |
| F101 | ICD-10 | F10.1 Harmful use |
| F102 | ICD-10 | F10.2 Dependence syndrome |
| F103 | ICD-10 | F10.3 Withdrawal state |
| F104 | ICD-10 | F10.4 Withdrawal state with delirium |
| F105 | ICD-10 | F10.5 Psychotic disorder |
| F106 | ICD-10 | F10.6 Amnesic syndrome |
| F107 | ICD-10 | F10.7 Residual and late-onset psychotic disorder |
| F108 | ICD-10 | F10.8 Other mental and behavioural disorders |
| F109 | ICD-10 | F10.9 Unspecified mental and behavioural disorder |
| G312 | ICD10 | G31.2 Degeneration of the nervous system due to alcohol |
| G621 | ICD10 | G62.1 Alcoholic polyneuropathy |
| G721 | ICD10 | G72.1 Alcoholic myopathy |
| I426 | ICD10 | I42.6 Alcoholic cardiomyopathy |
| K292 | ICD10 | K29.2 Alcoholic gastritis |
| T510 | ICD10 | T51.0 Toxic effect of ethanol |
| T519 | ICD10 | T51.9 Toxic effect of unspecified alcohol |
| X6509 | ICD-10 | X65.09 Intentional self-poisoning by and exposure to alcohol, Home, During unspecified activity |
| Y573 | ICD10 | Y57.3 Adverse effects in therapeutic use: alcohol deterrents |
| Z509 | ICD10 | Z50.2 Alcohol rehabilitation |
| Z714 | ICD10 | Z71.4 Alcohol abuse counselling and surveillance |
