# Supplementary material for: The impact of non-alcoholic fatty liver disease and liver fibrosis on adverse clinical outcomes and mortality in patients with chronic kidney disease: a prospective cohort study using the UK Biobank
Source: BMC Med. 2023 May 18;21:185. doi: 10.1186/s12916-023-02891-x (PMC10193672; doi:10.1186/s12916-023-02891-x)
Supplement: Supplementary file 4 — Additional file 4: Table S3. List of ICD-9 and ICD-10 codes used to define a cardiovascular event outcome and for the exclusion of a baseline cardiovascular event. [file 12916_2023_2891_MOESM4_ESM.docx]

**Supplementary Table 3** List of ICD-9 and ICD-10 codes used to define a cardiovascular event outcome and for the exclusion of a baseline cardiovascular event

| **UKBB code** | **Code type** | **Code** |
| --- | --- | --- |
| **Acute coronary syndrome** | | |
| NA | ICD9 | 410 Acute myocardial infarction |
| 4109 | ICD9 | 4109 Acute myocardial infarction |
| NA | ICD9 | 411 Other acute and subacute forms of ischemic heart disease |
| 4119 | ICD9 | 411.9 Other acute and subacute forms of ischemic heart disease |
| I200 | ICD10 | I20.0 Unstable angina |
| NA | ICD10 | I21 Acute myocardial infarction |
| I210 | ICD10 | 121.0 Acute transmural myocardial infarction of anterior wall |
| I211 | ICD10 | 121.1 Acute transmural myocardial infarction of inferior wall |
| I212 | ICD10 | 121.2 Acute transmural myocardial infarction of other sites |
| I213 | ICD10 | I21.3 Acute transmural myocardial infarction of unspecified site |
| I214 | ICD10 | I21.4 Acute subendocardial myocardial infarction |
| I219 | ICD10 | 121.5 Acute myocardial infarction, unspecified |
| I21X | ICD10 | I21.X Presumed acute myicardial infaction (unconfirmed) |
| NA | ICD10 | I22 Subsequent myocardial infarction |
| I220 | ICD10 | I22.0 Subsequent myocardial infarction of anterior wall |
| I221 | ICD10 | I22.1 Subsequent myocardial infarction of inferior wall |
| I228 | ICD10 | I22.8 Subsequent myocardial infarction of other sites |
| I229 | ICD10 | I22.9 Subsequent myocardial infarction of unspecified site |
| NA | ICD10 | I23 Certain current complications following acute myocardial infarction |
| I230 | ICD10 | I23.0 Haemopericardium as current complication following acute myocardial infarction |
| I231 | ICD10 | I23.1 Atrial septal defect as current complication following acute myocardial infarction |
| I232 | ICD10 | I23.2 Ventricular septal defect as current complication following acute myocardial infarction |
| I233 | ICD10 | I23.3 Rupture of cardiac wall without haemopericardium as current complication following acute myocardial infarction |
| I234 | ICD10 | I23.4 Rupture of chordae tendineae as current complication following acute myocardial infarction |
| I235 | ICD10 | I23.5 Rupture of papillary muscle as current complication following acute myocardial infarction |
| I236 | ICD10 | I23.6 Thrombosis of atrium, auricular appendage and ventricle as current complications following acute myocardial infarction |
| I238 | ICD10 | I23.8 Other current complications following acute myocardial infarction |
| **Cardiac failure** | | |
| NA | ICD9 | 428 Heart failure |
| 4280 | ICD9 | 428.0 Congestive heart failure |
| 4281 | ICD9 | 428.1 Left heart failure |
| 4289 | ICD9 | 428.9 Heart failure, unspecified |
| NA | ICD10 | I50 Heart failure |
| I500 | ICD10 | I50.0 Congestive heart failure |
| I501 | ICD10 | I50.1 Left ventricular failure |
| I509 | ICD10 | I50.9 Heart failure, unspecified |
| **Cardiac arrest** | | |
| 4275 | ICD9 | 427.5 Cardiac arrest |
| I460 | ICD10 | I46.0 Cardiac arrest with successful resuscitation |
| I469 | ICD10 | I46.9 Cardiac arrest, unspecified |
| **Cerebrovascular accident** | | |
| NA | ICD9 | 430 Subarachnoid hemorrhage |
| 4309 | ICD9 | 430.9 Subarachnoid hemorrhage |
| NA | ICD9 | 431 Intracerebral hemorrhage |
| 4319 | ICD9 | 431.9 Intracerebral haemorrhage |
| NA | ICD9 | 433 Occlusion and stenosis of precerebral arteries |
| 4330 | ICD9 | 433.0 Occlusion and stenosis of basilar artery |
| 4331 | ICD9 | 433.1 Occlusion and stenosis of carotid artery |
| 4332 | ICD9 | 433.2 Occlusion and stenosis of vertebral artery |
| 4333 | ICD9 | 433.3 Occlusion and stenosis of multiple and bilateral precerebral arteries |
| 4338 | ICD9 | 433.8 Occlusion and stenosis of other specified precerebral artery |
| 4339 | ICD9 | 433.9 Occlusion and stenosis of unspecified precerebral artery |
| NA | ICD9 | 434 Occlusion of cerebral arteries |
| 4340 | ICD9 | 434.0 Cerebral thrombosis |
| 4341 | ICD9 | 434.1 Cerebral embolism |
| 4349 | ICD9 | 434.9 Cerebral artery occlusion unspecified |
| NA | ICD9 | 435 Transient cerebral ischemia |
| 4359 | ICD9 | 4359 Transient cerebral ischaemia |
| 436 | ICD9 | 436 Acute but ill-defined cerebrovascular disease |
| 4369 | ICD9 | 4369 Acute but ill-defined cerebrovascular disease |
| 438 | ICD9 | 438 Late effects of cerebrovascular disease |
| 4389 | ICD9 | 4389 Late effects of cerebrovascular disease |
| NA | ICD10 | G45 Transient cerebral ischaemic attacks and related syndromes |
| G450 | ICD10 | G45.0 Vertebro-basilar artery syndrome |
| G451 | ICD10 | G45.1 Carotid artery syndrome (hemispheric) |
| G452 | ICD10 | G45.2 Multiple and bilateral precerebral artery syndromes |
| G453 | ICD10 | G45.3 Amaurosis fugax |
| G454 | ICD10 | G45.4 Transient global amnesia |
| G458 | ICD10 | G45.8 Other transient cerebral ischaemic attacks and related syndromes |
| G459 | ICD10 | G45.9 Transient cerebral ischaemic attack, unspecified |
| NA | ICD10 | G46 Vascular syndromes of brain in cerebrovascular diseases |
| G460 | ICD10 | G46.0 Middle cerebral artery syndrome |
| G461 | ICD10 | G46.1 Anterior cerebral artery syndrome |
| G462 | ICD10 | G46.2 Posterior cerebral artery syndrome |
| G463 | ICD10 | G46.3 Brain stem stroke syndrome |
| G464 | ICD10 | G46.4 Cerebellar stroke syndrome |
| G465 | ICD10 | G46.5 Pure motor lacunar syndrome |
| G466 | ICD10 | G46.6 Pure sensory lacunar syndrome |
| G467 | ICD10 | G46.7 Other lacunar syndromes |
| G468 | ICD10 | G46.8 Other vascular syndromes of brain in cerebrovascular diseases |
| NA | ICD10 | I60 Subarachnoid haemorrhage |
| I600 | ICD10 | I60.0 Subarachnoid haemorrhage from carotid siphon and bifurcation |
| I601 | ICD10 | I60.1 Subarachnoid haemorrhage from middle cerebral artery |
| I602 | ICD10 | I60.2 Subarachnoid haemorrhage from anterior communicating artery |
| I603 | ICD10 | I60.3 Subarachnoid haemorrhage from posterior communicating artery |
| I604 | ICD10 | I60.4 Subarachnoid haemorrhage from basilar artery |
| I605 | ICD10 | I60.5 Subarachnoid haemorrhage from vertebral artery |
| I606 | ICD10 | I60.6 Subarachnoid haemorrhage from other intracranial arteries |
| I607 | ICD10 | I60.7 Subarachnoid haemorrhage from intracranial artery, unspecified |
| I608 | ICD10 | I60.8 Other subarachnoid haemorrhage |
| I609 | ICD10 | I60.9 Subarachnoid haemorrhage, unspecified |
| NA | ICD10 | I61 Intracerebral haemorrhage |
| I610 | ICD10 | I61.0 Intracerebral haemorrhage in hemisphere, subcortical |
| I611 | ICD10 | I61.1 Intracerebral haemorrhage in hemisphere, cortical |
| I612 | ICD10 | I61.2 Intracerebral haemorrhage in hemisphere, unspecified |
| I613 | ICD10 | I61.3 Intracerebral haemorrhage in brain stem |
| I614 | ICD10 | I61.4 Intracerebral haemorrhage in cerebellum |
| I615 | ICD10 | I61.5 Intracerebral haemorrhage, intraventricular |
| I616 | ICD10 | I61.6 Intracerebral haemorrhage, multiple localised |
| I618 | ICD10 | I61.8 Other intracerebral haemorrhage |
| I619 | ICD10 | I61.9 Intracerebral haemorrhage, unspecified |
| NA | ICD10 | I63 Cerebral infarction |
| I630 | ICD10 | I63.0 Cerebral infarction due to thrombosis of precerebral arteries |
| I631 | ICD10 | I63.1 Cerebral infarction due to embolism of precerebral arteries |
| I632 | ICD10 | I63.2 Cerebral infarction due to unspecified occlusion or stenosis of precerebral arteries |
| I633 | ICD10 | I63.3 Cerebral infarction due to thrombosis of cerebral arteries |
| I634 | ICD10 | I63.4 Cerebral infarction due to embolism of cerebral arteries |
| I635 | ICD10 | I63.5 Cerebral infarction due to unspecified occlusion or stenosis of cerebral arteries |
| I636 | ICD10 | I63.6 Cerebral infarction due to cerebral venous thrombosis, nonpyogenic |
| I638 | ICD10 | I63.8 Other cerebral infarction |
| I639 | ICD10 | I63.9 Cerebral infarction, unspecified |
| I64 | ICD10 | I64 Stroke, not specified as haemorrhage or infarction |
| I690 | ICD10 | I69.0 Sequelae of subarachnoid haemorrhage |
| I691 | ICD10 | I69.1 Sequelae of intracerebral haemorrhage |
| I693 | ICD10 | I69.3 Sequelae of cerebral infarction |
| I694 | ICD10 | I69.4 Sequelae of stroke, not specified as haemorrhage or infarction |
| I720 | ICD10 | I72.0 Aneurysm of carotid artery |
| I725 | ICD10 | I72.5 Aneurysm and dissection of other precerebral arteries |
| I726 | ICD10 | I72.6 Aneurysm and dissection of vertebral artery |
| **Peripheral arterial disease** | | |
| 4410 | ICD9 | 4410 Dissecting aneurysm (any part) |
| 4411 | ICD9 | 4411 Thoracic aneurysm, ruptured |
| 4413 | ICD9 | 4413 Abdominal aneurysm, ruptured |
| 4415 | ICD9 | 4415 Aortic aneurysm of unspecified site, ruptured |
| 4438 | ICD9 | 4438 Other specified peripheral vascular disease |
| 4439 | ICD9 | 4439 Peripheral vascular disease, unspecified |
| NA | ICD9 | 444 Arterial embolism and thrombosis |
| 4440 | ICD9 | 4440 Embolism and thrombosis of abdominal aorta |
| 4441 | ICD9 | 4441 Embolism and thrombosis of other aorta |
| 4442 | ICD9 | 4442 Embolism and thrombosis of arteries of the extremities |
| 4448 | ICD9 | 4448 Embolism and thrombosis of other specified artery |
| 4449 | ICD9 | 4449 Embolism and thrombosis of unspecified artery |
| I710 | ICD10 | I71.0 Dissection of aorta [any part] |
| I711 | ICD10 | I71.1 Thoracic aortic aneurysm, ruptured |
| I713 | ICD10 | I71.3 Abdominal aortic aneurysm, ruptured |
| I715 | ICD10 | I71.5 Thoracoabdominal aortic aneurysm, ruptured |
| I718 | ICD10 | I71.8 Aortic aneurysm of unspecified site, ruptured |
| I738 | ICD10 | I73.8 Other specified peripheral vascular diseases |
| I739 | ICD10 | I73.9 Peripheral vascular disease, unspecified |
| NA | ICD10 | I74 Arterial embolism and thrombosis |
| I740 | ICD10 | I74.0 Embolism and thrombosis of abdominal aorta |
| I741 | ICD10 | I74.1 Embolism and thrombosis of other and unspecified parts of aorta |
| I742 | ICD10 | I74.2 Embolism and thrombosis of arteries of the upper extremities |
| I743 | ICD10 | I74.3 Embolism and thrombosis of arteries of the lower extremities |
| I744 | ICD10 | I74.4 Embolism and thrombosis of arteries of extremities, unspecified |
| I745 | ICD10 | I74.5 Embolism and thrombosis of iliac artery |
| I748 | ICD10 | I74.8 Embolism and thrombosis of other arteries |
| I749 | ICD10 | I74.9 Embolism and thrombosis of unspecified artery |
