# Supplementary material for: The impact of non-alcoholic fatty liver disease and liver fibrosis on adverse clinical outcomes and mortality in patients with chronic kidney disease: a prospective cohort study using the UK Biobank
Source: BMC Med. 2023 May 18;21:185. doi: 10.1186/s12916-023-02891-x (PMC10193672; doi:10.1186/s12916-023-02891-x)
Supplement: Supplementary file 5 — Additional file 5: Table S4. Number and proportion of patients in each Kidney Disease: Improving Global Outcomecategory according to baseline albuminuria and eGFR results. [file 12916_2023_2891_MOESM5_ESM.docx]

**Supplementary Table 4**. Number and proportion of patients in each Kidney Disease: Improving Global Outcome (KDIGO) category according to baseline albuminuria and eGFR results

a) All participants in the UK Biobank (with and without chronic kidney disease)

|  | **A1 (<3 mg/mmol), n (%)** | **A2 (3-30 mg/mmol),**  **n (%)** | **A3 (>30 mg/mmol), n (%)** | **Total,**  **n (%)** |
| --- | --- | --- | --- | --- |
| **G1 (≥90 ml/min/ 1.73m^2)^** | 228,671 (50.2) | 10,710 (2.4) | 573 (0.1) | 239,954 (52.7) |
| **G2 (60-89 ml/min/ 1.73m^2^)** | 192,745 (42.3) | 10,273 (2.3) | 856 (0.2) | 203,874 (44.8) |
| **G3a (45-59 ml/min/ 1.73m^2^)** | 7579 (1.7) | 1213 (0.3) | 291 (0.1) | 9083 (2.0) |
| **G3b (30-44 ml/min/ 1.73m^2^)** | 1078 (0.2) | 467 (0.1) | 194 (0.0) | 1739 (0.4) |
| **G4 (15-29 ml/min/ 1.73m^2^)** | 145 (0.0) | 141 (0.0) | 155 (0.0) | 441 (0.1) |
| **G5 (<15 ml/min/ 1.73m^2^)** | 4 (0.0) | 30 (0.0) | 49 (0.0) | 83 (0.0) |
| **Total** | 430,222 (94.5) | 22,834 (5.0) | 2118 (0.5) |  |

b) UK Biobank participants with chronic kidney disease meeting the study inclusion and exclusion criteria

|  | **A1 (<3 mg/mmol),**  **n (%)** | **A2 (3-30 mg/mmol),**  **n (%)** | **A3 (>30 mg/mmol),**  **n (%)** | **Total,**  **n (%)** |
| --- | --- | --- | --- | --- |
| **G1 (≥90 ml/min/ 1.73m^2^)** | 0 (0.0%) | 7114 (39.9%) | 411 (2.3%) | 7525 (42.2%) |
| **G2 (60-89 ml/min/ 1.73m^2^)** | 0 (0.0%) | 4943 (27.7%) | 465 (2.6%) | 5408 (30.3%) |
| **G3a (45-59 ml/min/ 1.73m^2^)** | 3160 (17.7%) | 569 (3.2%) | 169 (0.9%) | 3889 (21.8%) |
| **G3b (30-44 ml/min/ 1.73m^2^)** | 510 (2.9%) | 204 (1.1%) | 104 (0.6%) | 818 (4.6%) |
| **G4 (15-29 ml/min/ 1.73m^2^)** | 70 (0.4%) | 57 (0.3%) | 66 (0.4%) | 193 (1.1%) |
| **Total** | 3740 (21.0%) | 12,887 (72.3%) | 1206 (6.8%) |  |

Green: low risk of chronic kidney disease

Yellow: moderately increased risk of chronic kidney disease

Orange: high risk of chronic kidney disease

Red: very high risk of chronic kidney disease
