# Supplementary material for: The impact of non-alcoholic fatty liver disease and liver fibrosis on adverse clinical outcomes and mortality in patients with chronic kidney disease: a prospective cohort study using the UK Biobank
Source: BMC Med. 2023 May 18;21:185. doi: 10.1186/s12916-023-02891-x (PMC10193672; doi:10.1186/s12916-023-02891-x)
Supplement: Supplementary file 6 — Additional file 6: Table S5. Comparison of baseline demographics between participants excluded due to no data available to calculate HSI, FIB-4 and NFS and study cohort. [file 12916_2023_2891_MOESM6_ESM.docx]

**Supplementary Table 5.** Comparison of baseline demographics between participants excluded due to no data available to calculate HSI, FIB-4 and NFS and study cohort

|  | **Study cohort**  **(n=18,703)** | **Population excluded due to data unavailable to calculate HSI/FIB4/NFS (n=3761)** |
| --- | --- | --- |
| Median age, years (IQR) | 62 (12) | 61 (12) |
| ***Male (%)*** | 42.1 | 38.6 |
| ***Ethnicity (%)*** |  |  |
| White | 89.4 | 86.5 |
| Non-white | 10.0 | 12.8 |
| ***Townsend deprivation index*** |  |  |
| *Median score* | -1.55 | -1.32 |
| ***Alcohol*** |  |  |
| Weekly gram data available (%) | 72.7 | 75.4 |
| Mean alcohol grams per week | 40.0 | 33.7 |
| Non-drinkers (abstainers & former) (%) | 17.4 | 19.1 |
| ***Diabetes*** |  |  |
| Diabetes (%) | 19.5 | 20.6 |
| Median HbA1c people with diabetes,  mmol/mol | 54.7 | 55.0 |
| Median HbA1c overall, mmol/mol | 37.3 | 37.4 |
| ***Overweight/obesity*** |  |  |
| Median BMI, kg/m^2^ (IQR) | 28.6 (7.7) | 28.3 (8.0) |
| Weight categories |  |  |
| Overweight (BMI 25-30 kg/m^2^) (%) | 35.1 | 32.1 |
| Obese (BMI > 30 kg/m^2^) (%) | 40.6 | 36.9 |
| Median waist circumference, cm (IQR) | 95 (22) | 94 (22) |
| High risk (WC men > 102 cm, women > 88  cm) (%) | 48.3 | 47.6 |
| ***Lipids*** |  |  |
| Dyslipidaemia (%) | 72.0 | 56.0 |
| Median HDL (mmol/L) | 1.3 | 1.2 |
| Median TG (mmol/L) | 1.7 | 1.7 |
| ***Hypertensive (%)*** | 57.5 | 57.1 |
| ***Smoking (%)*** |  |  |
| Never smoked | 53.5 | 55.4 |
| Previous smoker | 33.9 | 31.1 |
| Current smoker | 11.6 | 12.2 |
| ***Liver enzymes*** |  |  |
| Median ALT (IU/L) | 21 | 20 |
| Median AST (IU/L) | 25 | 25 |
| Median GGT (IU/L) | 30 | 28 |
| Median platelets (10^6^/L) | 252 | 249 |
| Median albumin (g/L) | 45 | 45 |
| ***Baseline CVE (%)*** | 8.1 | 8.1 |
| ***Baseline eGFR*** |  |  |
| Median eGFR (ml/min/ 1.73m^2^) | 85 | 84 |
| G1 (≥ 90 ml/min/ 1.73m^2^) (%) | 41.6 | 28.2 |
| G2 (60-89 ml/min/ 1.73m^2^) (%) | 29.9 | 18.8 |
| G3a (45-59 ml/min/ 1.73m^2^) (%) | 22.4 | 16.1 |
| G3b (30-44 ml/min/ 1.73m^2^) (%) | 4.8 | 3.7 |
| G4 (15-29 ml/min/ 1.73m^2^) (%) | 1.2 | 1.4 |
| ***Baseline UACR*** |  |  |
| Median UACR (mg/mmol) | 39 | 40 |
| UACR < 3 mg/mmol (%) | 20.7 | 14.4 |
| UACR 3-30 mg/mmol (%) | 71.4 | 76.7 |
| UACR > 30 mg/mmol (%) | 6.7 | 7.8 |

NFS, Non-alcoholic fatty liver disease fibrosis score; FIB-4, fibrosis-4 score; AST aspartate transaminase; ALT, alanine transaminase; IQR, interquartile range; HbA1c, glycated haemoglobin; BMI, body mass index; WC, waist circumference; LDL, low density lipoprotein cholesterol; HDL, high density lipoprotein cholesterol; TG, triglycerides; ALT, alanine transaminase; AST, aspartate transaminase; GGT gamma glutamyl transferase; eGFR, estimated glomerular filtration rate; UACR, urine albumin creatinine ratio
