# Supplementary material for: The impact of non-alcoholic fatty liver disease and liver fibrosis on adverse clinical outcomes and mortality in patients with chronic kidney disease: a prospective cohort study using the UK Biobank
Source: BMC Med. 2023 May 18;21:185. doi: 10.1186/s12916-023-02891-x (PMC10193672; doi:10.1186/s12916-023-02891-x)
Supplement: Supplementary file 7 — Additional file 7: Table S6. Number and proportion of patients with NAFLD identified at baseline in each Kidney Disease: Improving Global Outcomecategory according to baseline albuminuria and eGFR results. [file 12916_2023_2891_MOESM7_ESM.docx]

**Supplementary Table 6**. Number and proportion of patients with NAFLD identified at baseline in each Kidney Disease: Improving Global Outcome (KDIGO) category according to baseline albuminuria and eGFR results

|  | **A1 (<3 mg/mmol), n (%)** | **A2 (3-30 mg/mmol),**  **n (%)** | **A3 (>30 mg/mmol), n (%)** | **Total,**  **n (%)** |
| --- | --- | --- | --- | --- |
| **G1 (≥90 ml/min/ 1.73m^2)^** | 0 (0) | 3406 (47.9) | 222 (54.0) | 3628 (48.2) |
| **G2 (60-89 ml/min/ 1.73m^2^)** | 0 (0) | 2930 (59.3) | 288 (61.9) | 3218 (59.5) |
| **G3a (45-59 ml/min/ 1.73m^2^)** | 2035 (64.4) | 364 (64.0) | 97 (57.4) | 2496 (64.2) |
| **G3b (30-44 ml/min/ 1.73m^2^)** | 338 (66.3) | 129 (63.2) | 54 (51.9) | 521 (63.7) |
| **G4 (15-29 ml/min/ 1.73m^2^)** | 39 (55.7) | 43 (75.4) | 33 (50.0) | 115 (59.6) |
| **Total** | 2412 (64.5) | 6872 (53.3) | 694 (57.5) |  |
