# Supplementary material for: The impact of non-alcoholic fatty liver disease and liver fibrosis on adverse clinical outcomes and mortality in patients with chronic kidney disease: a prospective cohort study using the UK Biobank
Source: BMC Med. 2023 May 18;21:185. doi: 10.1186/s12916-023-02891-x (PMC10193672; doi:10.1186/s12916-023-02891-x)
Supplement: Supplementary file 8 — Additional file 8: Table S7. Comparison of baseline demographics between participants with and without NAFLD fibrosis. [file 12916_2023_2891_MOESM8_ESM.docx]

**Supplementary Table 7.** Comparison of baseline demographics between participants with and without NAFLD fibrosis

|  | **NAFLD fibrosis score** | | | **Fibrosis-4 score** | | |
| --- | --- | --- | --- | --- | --- | --- |
|  | < -1.455 (< 0.12 if ≥ 65 yr) | - 1.455 - 0.676 (0.12-0.676 if ≥ 65 yr) | ≥ 0.676 | < 1.3 (<2.0 if ≥ 65 yrs) | 1.3-2.67 (2.0-2.67 if ≥ 65 yrs) | > 2.67 |
| ***N, (%)*** | 6057 (59.7) | 3311 (32.6) | 784 (7.7) | 7202 (70.9) | 2642 (26.0) | 308 (3.0) |
| ***Median age, years (IQR)*** | 62 (13) | 60 (8) | 64 (7) | 61 (13) | 62 (6) | 65 (6) |
| ***Male (%)*** | 39.4 | 49.9 | 56.8 | 40.3 | 52.8 | 59.1 |
| ***Ethnicity (%)*** |  |  |  |  |  |  |
| White | 90.4 | 86.7 | 87.2 | 89.0 | 88.7 | 89.3 |
| Non white | 9.1 | 12.7 | 11.5 | 10.5 | 10.5 | 9.7 |
| ***Townsend deprivation index*** |  |  |  |  |  |  |
| *Median score* | -1.54 | -0.68 | -0.12 | -1.19 | -1.17 | -1.29 |
| ***Alcohol*** |  |  |  |  |  |  |
| Weekly gram data available (%) | 70.4 | 71.5 | 73.5 | 69.6 | 74.1 | 76.3 |
| Mean alcohol grams per week | 40 | 27.7 | 8.1 | 34.0 | 34.0 | 28.0 |
| Non-drinkers (abstainers & former) (%) | 20.3 | 24.7 | 32.9 | 22.3 | 23.4 | 25.6 |
| ***Diabetes*** |  |  |  |  |  |  |
| Diabetes (%) | 16.1 | 46.2 | 77.7 | 30.3 | 30.8 | 38.6 |
| Median HbA1c people with diabetes, mmol/mol | 54.6 | 55.9 | 52.9 | 55.2 | 54.4 | 53.4 |
| Median HbA1c overall, mmol/mol | 37.8 | 41.8 | 49.7 | 39.0 | 39.1 | 40.3 |
| ***Overweight/obesity*** |  |  |  |  |  |  |
| Median BMI, kg/m^2^ (IQR) | 30.9 (4.7) | 34.0 (7.2) | 38.6 (9.2) | 32 (6.2) | 32.3 (6.1) | 33.6 (6.1) |
| Weight categories |  |  |  |  |  |  |
| Overweight (BMI 25-30 kg/m^2^) (%) | 37.2 | 16.1 | 3.4 | 29.5 | 24.6 | 12.7 |
| Obese (BMI > 30 kg/m^2^) (%) | 61.5 | 83.4 | 96.4 | 69.4 | 74.9 | 86.7 |
| Median waist circumference, cm (IQR) | 100 (15) | 108 (18) | 117.4 (19) | 102 (18) | 105 (17) | 109 (16) |
| High risk (WC men > 102 cm, women > 88 cm) (%) | 70.5% | 85.5% | 94.6% | 76.8% | 77.8% | 85.4% |
| ***Lipids*** |  |  |  |  |  |  |
| Dyslipidaemia (%) | 82.7 | 87 | 92.7 | 84.6 | 85.2 | 87.3 |
| Median HDL (mmol/L) | 1.2 | 1.1 | 1.0 | 1.2 | 1.1 | 1.1 |
| Median TG (mmol/L) | 2.0 | 2.0 | 2.0 | 2.0 | 2.0 | 2.0 |
| ***Hypertensive (%)*** | 62.1 | 74.4 | 88.9 | 66.3 | 72.7 | 75.6 |
| ***Smoking (%)*** |  |  |  |  |  |  |
| Never smoked | 53.4 | 48.4 | 42.2 | 51.8 | 48.9 | 47.4 |
| Previous smoker | 36.0 | 38.0 | 46.7 | 36.3 | 40.1 | 42.9 |
| Current smoker | 9.7 | 12.3 | 9.4 | 10.8 | 9.9 | 7.8 |
| ***Liver enzymes*** |  |  |  |  |  |  |
| Median ALT (IU/L) | 25 | 24 | 21 | 24 | 26 | 30 |
| Median AST (IU/L) | 25 | 26 | 26 | 24 | 30 | 40 |
| Median GGT (IU/L) | 35 | 35 | 37 | 34 | 38 | 49 |
| Median platelets (10^6^/L) | 277 | 227 | 189 | 274 | 206 | 146 |
| Median albumin (g/L) | 45 | 44 | 43 | 45 | 45 | 44 |
| ***Baseline CVE (%)*** | 7.3 | 10.9 | 19.4 | 8.4 | 11.5 | 16.2 |
| ***Baseline eGFR*** |  |  |  |  |  |  |
| Median eGFR (ml/min/ 1.73m^2^) | 83 | 79 | 60 | 83 | 76 | 67 |
| G1 (≥ 90 ml/min/ 1.73m^2^) (%) | 39.6 | 32.9 | 18.0 | 38.8 | 29.0 | 21.8 |
| G2 (60-89 ml/min/ 1.73m^2^) (%) | 30.7 | 33.7 | 31.0 | 30.4 | 35.1 | 31.8 |
| G3a (45-59 ml/min/ 1.73m^2^) (%) | 24.8 | 25.7 | 33.8 | 24.5 | 27.9 | 39.6 |
| G3b (30-44 ml/min/ 1.73m^2^) (%) | 4.1 | 6.1 | 12.9 | 5.1 | 6.6 | 4.5 |
| G4 (15-29 ml/min/ 1.73m^2^) (%) | 0.7 | 1.7 | 4.0 | 1.2 | 1.3 | 2.3 |
| ***Baseline UACR*** |  |  |  |  |  |  |
| Median UACR (mg/mmol) | 40 | 52 | 56 | 43 | 47 | 45 |
| UACR < 3 mg/mmol (%) | 22.8 | 23.7 | 31.1 | 22.6 | 26.1 | 30.2 |
| UACR 3-30 mg/mmol (%) | 70.4 | 66.0 | 54.5 | 69.1 | 65.0 | 61.0 |
| UACR > 30 mg/mmol (%) | 5.4 | 8.4 | 11.5 | 6.6 | 7.5 | 7.1 |

NAFLD, non-alcoholic fatty liver disease; IQR, interquartile range; HbA1c, glycated haemoglobin; BMI, body mass index; WC, waist circumference; LDL, low density lipoprotein cholesterol; HDL, high density lipoprotein cholesterol; TG, triglycerides; ALT, alanine transaminase; AST, aspartate transaminase; GGT gamma glutamyl transferase; eGFR, estimated glomerular filtration rate; UACR, urine albumin creatinine ratio
