# Supplementary material for: The impact of non-alcoholic fatty liver disease and liver fibrosis on adverse clinical outcomes and mortality in patients with chronic kidney disease: a prospective cohort study using the UK Biobank
Source: BMC Med. 2023 May 18;21:185. doi: 10.1186/s12916-023-02891-x (PMC10193672; doi:10.1186/s12916-023-02891-x)
Supplement: Supplementary file 9 — Additional file 9: Figure S1. Primary outcome event rates for individuals with and without NAFLD. [file 12916_2023_2891_MOESM9_ESM.docx]

**Supplementary Figure 1.** Primary outcome event rates for individuals with and without NAFLD

NAFLD, non-alcoholic fatty liver disease; CVE, cardiovascular event; ACS, acute coronary syndrome; HF, heart failure; CVA, cerebrovascular accident; PAD, peripheral arterial disease; ESRD, end-stage renal disease
