# Supplementary material for: The impact of non-alcoholic fatty liver disease and liver fibrosis on adverse clinical outcomes and mortality in patients with chronic kidney disease: a prospective cohort study using the UK Biobank
Source: BMC Med. 2023 May 18;21:185. doi: 10.1186/s12916-023-02891-x (PMC10193672; doi:10.1186/s12916-023-02891-x)
Supplement: Supplementary file 10 — Additional file 10: Table S8. Univariate analysis of factors predictive of primary outcomes for people with CKD. [file 12916_2023_2891_MOESM10_ESM.docx]

**Supplementary Table 8.** Univariate analysis of factors predictive of primary outcomes for people with CKD

|  | **Cardiovascular events, HR (95% CI)** | | | | | **End-stage renal disease, HR (95% CI)** | **All-cause mortality, HR (95% CI)** |
| --- | --- | --- | --- | --- | --- | --- | --- |
|  | **All cardiovascular events** | **Acute coronary syndrome** | **Heart failure** | **Cerebrovascular accident** | **Peripheral arterial disease** |  |  |
| n | n=1320 | n=468 | n=538 | n=471 | n=277 | n=157 | n=1489 |
| *Age (years)* |  |  |  |  |  |  |  |
| < 45 | 1.00 Ref. | 1.00 Ref. | 1.00 Ref. | 1.00 Ref. | 1.00 Ref. | 1.00 Ref. | 1.00 Ref. |
| 45-54 | 2.52 (1.85-3.43) **** | 1.92 (1.21-3.06) ** | 2.57 (1.57-4.2) *** | 2.86 (1.67-4.87) *** | 1.59 (0.83-3.05) p = 0.1595 | 1.04 (0.68-1.61) | 3.53 (2.36-5.28) **** |
| 55-64 | 4.81 (3.58-6.46) **** | 3.47 (2.24-5.38) **** | 6.11 (3.82-9.76) **** | 5.02 (3-8.4) **** | 3.97 (2.17-7.25) **** | 1.01 (0.68-1.52) | 7.64 (5.18-11.27) **** |
| >65 | 7.71 (5.75-10.34) **** | 4.79 (3.09-7.42) **** | 10.55 (6.62-16.83) **** | 7.78 (4.66-12.99) **** | 6.35 (3.48-11.59) **** | 0.83 (0.54-1.26) | 13.92 (9.45-20.5) **** |
| *Gender* |  |  |  |  |  |  |  |
| Female | 1.00 Ref. | 1.00 Ref. | 1.00 Ref. | 1.00 Ref. | 1.00 Ref. | 1.00 Ref. | 1.00 Ref. |
| Male | 1.70 (1.62-1.79) **** | 1.95 (1.78-2.13) **** | 1.74 (1.63-1.87) **** | 1.57 (1.44-1.7) **** | 1.83 (1.64-2.05) **** | 1.85 (1.6-2.14) **** | 1.63 (1.55-1.72) **** |
| *Ethnicity* |  |  |  |  |  |  |  |
| White | 1.00 Ref. | 1.00 Ref. | 1.00 Ref. | 1.00 Ref. | 1.00 Ref. | 1.00 Ref. | 1.00 Ref. |
| Non-white | 0.82 (0.73-0.93) ** | 0.9 (0.73-1.11) | 0.8 (0.67-0.95) * | 0.9 (0.74-1.1) | 0.64 (0.47-0.87) p = 0.004 | 1.84 (1.41-2.39) **** | 0.57 (0.5-0.66) **** |
| *Townsend deprivation index* |  |  |  |  |  |  |  |
| Least deprived quartile | 1.00 Ref. | 1.00 Ref. | 1.00 Ref. | 1.00 Ref. | 1.00 Ref. | 1.00 Ref. | 1.00 Ref. |
| 2^nd^ quartile | 1.03 (0.92-1.15) | 1 (0.82-1.21) | 1.02 (0.87-1.19) | 1.08 (0.9-1.31) | 1.21 (0.92-1.59) | 0.95 (0.67-1.36) | 1.04 (0.93-1.17) |
| 3^rd^ quartile | 1.13 (1.01-1.27) * | 1.07 (0.89-1.29) | 1.19 (1.03-1.39) * | 1.15 (0.96-1.38) | 1.48 (1.15-1.92) ** | 1.4 (1.02-1.93) * | 1.2 (1.07-1.34) ** |
| Most deprived quartile | 1.39 (1.25-1.54) **** | 1.3 (1.09-1.56) ** | 1.49 (1.29-1.71) **** | 1.33 (1.12-1.58) ** | 2.01 (1.58-2.55) **** | 1.77 (1.31-2.39) *** | 1.53 (1.38-1.7) **** |
| *Diabetes* |  |  |  |  |  |  |  |
| No diabetes | 1.00 Ref. | 1.00 Ref. | 1.00 Ref. | 1.00 Ref. | 1.00 Ref. | 1.00 Ref. | 1.00 Ref. |
| Diabetes | 2.3 (2.13-2.49) **** | 2.66 (2.34-3.03) **** | 2.75 (2.49-3.04) **** | 2.14 (1.89-2.43) **** | 3.2 (2.74-3.74) **** | 3.23 (2.64-3.96) **** | 2.18 (2.02-2.34) **** |
| *BMI (kg/m^2^)* |  |  |  |  |  |  |  |
| BMI < 25 | 1.00 Ref. | 1.00 Ref. | 1.00 Ref. | 1.00 Ref. | 1.00 Ref. | 1.00 Ref. | 1.00 Ref. |
| BMI 25-30 | 0.7 (0.63-0.78) **** | 0.6 (0.5-0.73) **** | 0.72 (0.62-0.84) **** | 0.69 (0.58-0.81) **** | 0.8 (0.64-1.0) * | 0.76 (0.57-1.01) | 0.89 (0.81-0.98) * |
| BMI > 30 | 1.34 (1.24-1.46) **** | 1.28 (1.11-1.46) *** | 1.72 (1.54-1.92) **** | 1.05 (0.92-1.2) | 1.31 (1.1-1.56) ** | 1.2 (0.96-1.5) | 1.26 (1.16-1.36) **** |
| *Waist circumference (cm)* |  |  |  |  |  |  |  |
| M < 94, W < 80 | 1.00 Ref. | 1.00 Ref. | 1.00 Ref. | 1.00 Ref. | 1.00 Ref. | 1.00 Ref. | 1.00 Ref. |
| M 94-102, W 80-88 | 1.26 (1.12-1.4) *** | 1.40 (1.16-1.69) *** | 1.26 (1.08-1.48) ** | 1.15 (0.97-1.37) | 1.22 (0.96-1.56) | 1.18 (0.88-1.58) | 1.07 (0.96-1.19) |
| M > 102, W > 88 | 1.78 (1.62-1.96) **** | 1.82 (1.55-2.15) **** | 2.11 (1.85-2.41) **** | 1.43 (1.24-1.66) **** | 1.85 (1.51-2.26) **** | 1.38 (1.07-1.77) * | 1.49 (1.37-1.63) **** |
| *Hypertension* |  |  |  |  |  |  |  |
| No hypertension | 1.00 Ref. | 1.00 Ref. | 1.00 Ref. | 1.00 Ref. | 1.00 Ref. | 1.00 Ref. | 1.00 Ref. |
| Hypertension | 2.81 (2.58-3.05) **** | 2.85 (2.46-3.29) **** | 4.57 (4-5.22) **** | 2.15 (1.88-2.45) **** | 4.8 (3.86-5.98) **** | 5.96 (4.37-8.14) **** | 2.17 (2.01-2.35) **** |
| *Dyslipidaemia* |  |  |  |  |  |  |  |
| No dyslipidaemia | 1.00 Ref. | 1.00 Ref. | 1.00 Ref. | 1.00 Ref. | 1.00 Ref. | 1.00 Ref. | 1.00 Ref. |
| Dyslipidaemia | 2.09 (1.91-2.3) **** | 2.83 (2.36-3.39) **** | 2.8 (2.42-3.23) **** | 1.67 (1.44-1.93) **** | 2.79 (2.22-3.51) **** | 3.16 (2.31-4.32) **** | 1.88 (1.72-2.06) **** |
| *Alcohol (u/week)* |  |  |  |  |  |  |  |
| Low risk drinkers | 1.00 Ref. | 1.00 Ref. | 1.00 Ref. | 1.00 Ref. | 1.00 Ref. | 1.00 Ref. | 1.00 Ref. |
| Never drinkers | 1.15 (1.02-1.3) * | 1.23 (1-1.5) * | 1.1 (0.93-1.3) | 1.29 (1.06-1.56) * | 1.28 (0.99-1.65) | 1.23 (0.89-1.71) | 1.17 (1.04-1.31) ** |
| Former drinkers | 1.64 (1.45-1.85) **** | 1.66 (1.35-2.04) **** | 1.76 (1.5-2.06) **** | 1.65 (1.36-2.01) **** | 2.15 (1.7-2.72) p = <0.0001**** | 1.86 (1.36-2.55) *** | 1.88 (1.69-2.1) **** |
| *Smoking* |  |  |  |  |  |  |  |
| Never smoker | 1.00 Ref. | 1.00 Ref. | 1.00 Ref. | 1.00 Ref. | 1.00 Ref. | 1.00 Ref. | 1.00 Ref. |
| Previous smoker | 1.61 (1.49-1.75) **** | 1.73 (1.51-1.98) **** | 1.82 (1.64-2.02) **** | 1.39 (1.22-1.58) **** | 2.16 (1.8-2.58) **** | 1.26 (1.01-1.56) * | 1.89 (1.75-2.05) **** |
| Current smoker | 1.98 (1.79-2.2) **** | 1.97 (1.65-2.36) **** | 1.82 (1.57-2.11) **** | 1.84 (1.55-2.18) **** | 3.91 (3.18-4.8) **** | 1.28 (0.93-1.75) | 2.48 (2.25-2.74) **** |
| *Baseline eGFR (ml/min/ 1.73m^2^)* |  |  |  |  |  |  |  |
| eGFR > 45 | 1.00 Ref. | 1.00 Ref. | 1.00 Ref. | 1.00 Ref. | 1.00 Ref. | 1.00 Ref. | 1.00 Ref. |
| eGFR < 45 | 2.71 (2.4-3.05) **** | 2.53 (2.09-3.07) **** | 3.56 (3.1-4.09) **** | 1.8 (1.46-2.23) **** | 3.18 (2.55-3.97) **** | 29.63 (24.16-36.33) **** | 3.1 (2.81-3.43) **** |
| *Baseline UACR (mg/mmol)* |  |  |  |  |  |  |  |
| UACR < 3 | 1.0 Ref. | Ref. | 1.00 Ref. | 1.00 Ref. | 1.00 Ref. | 1.00 Ref. | 1.00 Ref. |
| UACR 3 – 30 † | 0.59 (0.54-0.64) **** | 0.63 (0.55-0.73) **** | 0.49 (0.44-0.55) **** | 0.74 (0.65-0.86) **** | 0.57 (0.48-0.68) **** | 0.5 (0.37-0.66) **** | 0.54 (0.5-0.58) **** |
| UACR > 30 | 1.16 (1.02-1.33) * | 1.32 (1.06-1.65) * | 1.23 (1.04-1.45) * | 1.31 (1.05-1.64) * | 1.42 (1.09-1.85) ** | 7.98 (6.04-10.55) **** | 1.1 (0.97-1.25) |

* p<0.05, ** p<0.01, *** p<0.001, **** p <0.0001

† Results shown for individuals with eGFR < 60 ml/min/ 1.73m^2^ only

HR, hazard ratio; CI, confidence interval; BMI, body mass index; eGFR, estimated glomerular filtration rate; UACR, urine albumin creatinine ratio

Low risk drinkers: > 0-112 grams / week (equivalent in UK > 0-14 units / week)
