# Supplementary material for: The impact of non-alcoholic fatty liver disease and liver fibrosis on adverse clinical outcomes and mortality in patients with chronic kidney disease: a prospective cohort study using the UK Biobank
Source: BMC Med. 2023 May 18;21:185. doi: 10.1186/s12916-023-02891-x (PMC10193672; doi:10.1186/s12916-023-02891-x)
Supplement: Supplementary file 11 — Additional file 11: Table S9. Event ratesof primary outcome events according to baseline Kidney Disease: Improving Global Outcomecategory. [file 12916_2023_2891_MOESM11_ESM.docx]

**Supplementary Table 9**. Event rates (per person year) of primary outcome events according to baseline Kidney Disease: Improving Global Outcome (KDIGO) category

1. All cardiovascular events

|  | **A1 (<3 mg/mmol)** | **A2 (3-30 mg/mmol)** | **A3 (>30 mg/mmol)** |
| --- | --- | --- | --- |
| **G1 (≥90 ml/min/ 1.73m^2)^** | NA | 0.008 | 0.015 |
| **G2 (60-89 ml/min/ 1.73m^2^)** | NA | 0.017 | 0.025 |
| **G3a (45-59 ml/min/ 1.73m^2^)** | 0.019 | 0.028 | 0.037 |
| **G3b (30-44 ml/min/ 1.73m^2^)** | 0.031 | 0.033 | 0.039 |
| **G4 (15-29 ml/min/ 1.73m^2^)** | 0.049 | 0.051 | 0.067 |

1. End-stage renal disease

|  | **A1 (<3 mg/mmol)** | **A2 (3-30 mg/mmol)** | **A3 (>30 mg/mmol)** |
| --- | --- | --- | --- |
| **G1 (≥90 ml/min/ 1.73m^2)^** | NA | 0 | 0.001 |
| **G2 (60-89 ml/min/ 1.73m^2^)** | NA | 0 | 0.005 |
| **G3a (45-59 ml/min/ 1.73m^2^)** | 0.001 | 0.003 | 0.02 |
| **G3b (30-44 ml/min/ 1.73m^2^)** | 0.005 | 0.016 | 0.052 |
| **G4 (15-29 ml/min/ 1.73m^2^)** | 0.022 | 0.076 | 0.153 |

1. All-cause mortality

|  | **A1 (<3 mg/mmol)** | **A2 (3-30 mg/mmol)** | **A3 (>30 mg/mmol)** |
| --- | --- | --- | --- |
| **G1 (≥90 ml/min/ 1.73m^2)^** | NA | 0.006 | 0.01 |
| **G2 (60-89 ml/min/ 1.73m^2^)** | NA | 0.015 | 0.023 |
| **G3a (45-59 ml/min/ 1.73m^2^)** | 0.018 | 0.029 | 0.034 |
| **G3b (30-44 ml/min/ 1.73m^2^)** | 0.028 | 0.047 | 0.038 |
| **G4 (15-29 ml/min/ 1.73m^2^)** | 0.051 | 0.056 | 0.061 |
