# Supplementary material for: The impact of non-alcoholic fatty liver disease and liver fibrosis on adverse clinical outcomes and mortality in patients with chronic kidney disease: a prospective cohort study using the UK Biobank
Source: BMC Med. 2023 May 18;21:185. doi: 10.1186/s12916-023-02891-x (PMC10193672; doi:10.1186/s12916-023-02891-x)
Supplement: Supplementary file 12 — Additional file 12: Table S10. Sensitivity analysis showing the association of NAFLD with CVEs, ESRD and all-cause mortality where eGFR was calculated using serum creatinine alone. [file 12916_2023_2891_MOESM12_ESM.docx]

**Supplementary Table 10.** Sensitivity analysis showing the association of NAFLD with CVEs, ESRD and all-cause mortality where eGFR was calculated using serum creatinine alone (n=17,689 people with CKD in total)

|  | **Cardiovascular events, HR (95% CI)** | | | | | **End-stage renal disease, HR (95% CI)** | **All-cause mortality, HR (95% CI)** |
| --- | --- | --- | --- | --- | --- | --- | --- |
|  | **All cardiovascular events** | **Acute coronary syndrome** | **Heart failure** | **Cerebrovascular accident** | **Peripheral arterial disease** |  |  |
| **Univariate model** | | | | | | | |
| No NAFLD | 1.00 Ref. | 1.00 Ref. | 1.00 Ref. | 1.00 Ref. | 1.00 Ref. | 1.00 Ref. | 1.00 Ref. |
| NAFLD | 1.51 (1.4-1.63) **** | 1.68 (1.47-1.92) **** | 1.78 (1.59-1.98) **** | 1.35 (1.19-1.53) **** | 1.48 (1.25-1.75) **** | 1.34 (1.09-1.64) ** | 1.24 (1.15-1.34) **** |
| NFS Low risk | 1.00 Ref. | 1.00 Ref. | 1.00 Ref. | 1.00 Ref. | 1.00 Ref. | 1.00 Ref. | 1.00 Ref. |
| NFS Intermediate risk | 1.42 (1.28-1.57) **** | 1.46 (1.24-1.73) **** | 1.61 (1.41-1.85) **** | 1.24 (1.04-1.47) * | 1.53 (1.23-1.91) *** | 3.13 (2.34-4.18) **** | 1.29 (1.16-1.43) **** |
| NFS High risk | 2.50 (2.14-2.92) **** | 2.05 (1.57-2.67) **** | 3.62 (3.02-4.34) **** | 2.18 (1.69-2.80) **** | 2.81 (2.06-3.84) **** | 5.40 (3.69-7.91) **** | 2.75 (2.39-3.17) **** |
| FIB-4 Low risk | 1.00 Ref. | 1.00 Ref. | 1.00 Ref. | 1.00 Ref. | 1.00 Ref. | 1.00 Ref. | 1.00 Ref. |
| FIB-4 Intermediate risk | 1.23 (1.11-1.36) *** | 1.15 (0.97-1.38) | 1.15 (1.00-1.32) | 1.14 (0.96-1.36) | 1.23 (0.99-1.55) | 1.42 (1.08-1.87) * | 1.03 (0.92-1.15) |
| FIB-4 High risk | 1.56 (1.20-2.03) *** | 1.30 (0.83-2.04) | 1.83 (1.34-2.50) *** | 1.68 (1.13-2.49) * | 1.41 (0.81-2.46) | 1.45 (0.71-2.94) | 1.69 (1.34-2.14) **** |
| **Multivariate model**† | | | | | | | |
| No NAFLD | 1.00 Ref. | 1.00 Ref. | 1.00 Ref. | 1.00 Ref. | 1.00 Ref. | 1.00 Ref. | 1.00 Ref. |
| NAFLD | 1.25 (1.15-1.36) **** | 1.27 (1.09-1.47) ** | 1.35 (1.20-1.52) **** | 1.10 (0.96-1.27) | 1.04 (0.86-1.26) | 0.92 (0.72-1.16) | 0.96 (0.88-1.04) |
| NFS Low risk | 1.00 Ref. | 1.00 Ref. | 1.00 Ref. | 1.00 Ref. | 1.00 Ref. | 1.00 Ref. | 1.00 Ref. |
| NFS Intermediate risk | 1.15 (1.03-1.29) * | 1.02 (0.84-1.22) | 1.32 (1.13-1.54) *** | 1.01 (0.84-1.23) | 1.04 (0.81-1.34) | 1.32 (0.95-1.83) | 1.04 (0.92-1.16) |
| NFS High risk | 1.18 (0.99-1.41) | 0.80 (0.59-1.07) | 1.66 (1.35-2.04) **** | 1.07 (0.80-1.42) | 1.04 (0.73-1.48) | 1.06 (0.67-1.67) | 1.19 (1.01-1.40) * |
| FIB-4 Low risk | 1.00 Ref. | 1.00 Ref. | 1.00 Ref. | 1.00 Ref. | 1.00 Ref. | 1.00 Ref. | 1.00 Ref. |
| FIB-4 Intermediate risk | 1.09 (0.98-1.21) | 1.00 (0.83-1.19) | 1.03 (0.89-1.19) | 1.05 (0.88-1.26) | 1.08 (0.86-1.36) | 1.04 (0.77-1.41) | 0.90 (0.80-1.01) |
| FIB-4 High risk | 0.95 (0.73-1.25) | 0.84 (0.53-1.34) | 1.17 (0.86-1.61) | 1.06 (0.70-1.60) | 0.90 (0.51-1.57) p | 0.93 (0.45-1.92) p | 1.06 (0.83-1.36) |

* p<0.05, ** p<0.01, *** p<0.001, **** p <0.0001

† Adjusted for age, sex, deprivation, ethnicity, alcohol (continuous), smoking, baseline eGFR and UACR, diabetes

NAFLD, Non-alcoholic fatty liver disease; HR, hazard ratio; CI, confidence interval
