# Supplementary material for: The impact of non-alcoholic fatty liver disease and liver fibrosis on adverse clinical outcomes and mortality in patients with chronic kidney disease: a prospective cohort study using the UK Biobank
Source: BMC Med. 2023 May 18;21:185. doi: 10.1186/s12916-023-02891-x (PMC10193672; doi:10.1186/s12916-023-02891-x)
Supplement: Supplementary file 13 — Additional file 13: Table S11. Sensitivity analysis showing the association of NAFLD with CVEs, ESRD and all-cause mortality where CKD is defined according toeGFR < 60 ml/min/1.73m2 alone,albuminuria > 3mg/mmol alone, andfor patients meeting both criteria. [file 12916_2023_2891_MOESM13_ESM.docx]

**Supplementary Table 11.** Sensitivity analysis showing the association of NAFLD with CVEs, ESRD and all-cause mortality where CKD is defined according to (i) eGFR < 60 ml/min/1.73m^2^ alone, (ii) albuminuria > 3mg/mmol alone, and (iii) for patients meeting both criteria.

1. **CKD definition:** eGFR < 60 ml/min/1.73m^2^ (n=5128)

|  | **Cardiovascular events, HR (95% CI)** | | | | | **End-stage renal disease, HR (95% CI)** | **All-cause mortality, HR (95% CI)** |
| --- | --- | --- | --- | --- | --- | --- | --- |
|  | **All cardiovascular events** | **Acute coronary syndrome** | **Heart failure** | **Cerebrovascular accident** | **Peripheral arterial disease** |  |  |
| **Univariate model** | | | | | | | |
| No NAFLD | 1.00 Ref. | 1.00 Ref. | 1.00 Ref. | 1.00 Ref. | 1.00 Ref. | 1.00 Ref. | 1.00 Ref. |
| NAFLD | 1.25 (1.1-1.42) *** | 1.21 (0.98-1.49) | 1.41 (1.2-1.65) **** | 1.07 (0.87-1.32) | 1.1 (0.86-1.4) | 0.86 (0.68-1.09) | 0.93 (0.84-1.03) |
| **Multivariate model**† | | | | | | | |
| No NAFLD | 1.00 Ref. | 1.00 Ref. | 1.00 Ref. | 1.00 Ref. | 1.00 Ref. | 1.00 Ref. | 1.00 Ref. |
| NAFLD | 1.22 (1.06-1.40) ** | 1.21 (0.95-1.53) | 1.30 (1.09-1.55) ** | 0.98 (0.77-1.24) | 1.00 (0.76-1.31) | 0.83 (0.63-1.10) | 0.85 (0.75-0.95) ** |

1. **CKD definition:** albuminuria > 3mg/mmol (n=14,105)

|  | **Cardiovascular events, HR (95% CI)** | | | | | **End-stage renal disease, HR (95% CI)** | **All-cause mortality, HR (95% CI)** |
| --- | --- | --- | --- | --- | --- | --- | --- |
|  | **All cardiovascular events** | **Acute coronary syndrome** | **Heart failure** | **Cerebrovascular accident** | **Peripheral arterial disease** |  |  |
| **Univariate model** | | | | | | | |
| No NAFLD | 1.00 Ref. | 1.00 Ref. | 1.00 Ref. | 1.00 Ref. | 1.00 Ref. | 1.00 Ref. | 1.00 Ref. |
| NAFLD | 1.49 (1.36-1.62) **** | 1.64 (1.41-1.91) **** | 1.74 (1.54-1.97) **** | 1.37 (1.2-1.58) **** | 1.61 (1.33-1.96) **** | 1.32 (1.05-1.67) * | 1.29 (1.19-1.41) **** |
| **Multivariate model**† | | | | | | | |
| No NAFLD | 1.00 Ref. | 1.00 Ref. | 1.00 Ref. | 1.00 Ref. | 1.00 Ref. | 1.00 Ref. | 1.00 Ref. |
| NAFLD | 1.16 (1.05-1.28) ** | 1.17 (0.99-1.38) | 1.21 (1.06-1.39) ** | 1.08 (0.93-1.26) | 1.03 (0.83-1.28) | 0.73 (0.56-0.96) * | 0.92 (0.84-1.01) |

1. **CKD definition:** eGFR < 60 ml/min/1.73m^2^ and albuminuria > 3mg/mmol (n=1066)

|  | **Cardiovascular events, HR (95% CI)** | | | | | **End-stage renal disease, HR (95% CI)** | **All-cause mortality, HR (95% CI)** |
| --- | --- | --- | --- | --- | --- | --- | --- |
|  | **All cardiovascular events** | **Acute coronary syndrome** | **Heart failure** | **Cerebrovascular accident** | **Peripheral arterial disease** |  |  |
| **Univariate model** | | | | | | | |
| No NAFLD | 1.00 Ref. | 1.00 Ref. | 1.00 Ref. | 1.00 Ref. | 1.00 Ref. | 1.00 Ref. | 1.00 Ref. |
| NAFLD | 1.35 (1.07-1.7) * | 1.11 (0.76-1.62) | 1.42 (1.07-1.89) * | 1.28 (0.86-1.91) | 1.36 (0.89-2.06) | 0.9 (0.69-1.19) | 1.06 (0.88-1.28) |
| **Multivariate model**† | | | | | | | |
| No NAFLD | 1.00 Ref. | 1.00 Ref. | 1.00 Ref. | 1.00 Ref. | 1.00 Ref. | 1.00 Ref. | 1.00 Ref. |
| NAFLD | 1.00 (0.77-1.30) | 0.95 (0.62-1.46) | 0.97 (0.70-1.34) | 0.97 (0.62-1.52) | 0.88 (0.54-1.42) | 0.81 (0.59-1.12) | 0.70 (0.56-0.87) ** |

* p<0.05, ** p<0.01, *** p<0.001, **** p <0.0001

† Adjusted for age, sex, deprivation, ethnicity, smoking, baseline eGFR and UACR, diabetes

NAFLD, Non-alcoholic fatty liver disease; HR, hazard ratio; CI, confidence interval

**Supplementary Figure 1.** Primary outcome event rates for individuals with and without NAFLD

NAFLD, non-alcoholic fatty liver disease; CVE, cardiovascular event; ACS, acute coronary syndrome; HF, heart failure; CVA, cerebrovascular accident; PAD, peripheral arterial disease; ESRD, end-stage renal disease
